# Synthesis, Anticancer Assessment, and Molecular Docking of Novel Chalcone-Thienopyrimidine Derivatives in HepG2 and MCF-7 Cell Lines

**DOI:** 10.1155/2021/4759821

**Published:** 2021-12-28

**Authors:** Ghada M. Safwat, Kamel M. A. Hassanin, Eman T. Mohammed, Essam Kh. Ahmed, Mahmoud R. Abdel Rheim, Mohamed A. Ameen, Mohamed Abdel-Aziz, Ahmed M. Gouda, Ilaria Peluso, Rafa Almeer, Mohamed M. Abdel-Daim, Ahmed Abdel-Wahab

**Affiliations:** ^1^Department of Biochemistry, Faculty of Veterinary Medicine, Beni Suef University, Beni Suef 62512, Egypt; ^2^Biochemistry Department, Faculty of Veterinary Medicine, Minia University, Minya 61519, Egypt; ^3^Chemistry Department, Faculty of Science, Minia University, Minya 61519, Egypt; ^4^Medicinal Chemistry Department, Faculty of Pharmacy, Minia University, Minya 61519, Egypt; ^5^Medicinal Chemistry Department, Faculty of Pharmacy, Beni Suef University, Beni Suef 62514, Egypt; ^6^Research Centre for Food and Nutrition, Council for Agricultural Research and Economics (CREA-AN), Rome, Italy; ^7^Department of Zoology, College of Science, King Saud University, P.O. Box 2455, Riyadh 11451, Saudi Arabia; ^8^Pharmacology Department, Faculty of Veterinary Medicine, Suez Canal University, Ismailia 41522, Egypt; ^9^Physiology Department, Faculty of Veterinary Medicine, Minia University, Minya 61519, Egypt

## Abstract

Heterocycles containing thienopyrimidine moieties have attracted attention due to their interesting biological and pharmacological activities. In this research article, we reported the synthesis of a series of new hybrid molecules through merging the structural features of chalcones and pyridothienopyrimidinones. Our results indicated that the synthesis of chalcone-thienopyrimidine derivatives from the corresponding thienopyrimidine and chalcones proceeded in a relatively short reaction time with good yields and high purity. Most of these novel compounds exhibited moderate to robust cytotoxicity against HepG2 and MCF-7 cancer cells similar to that of 5-fluorouracil (5-FU). The results indicated that IC_50_ of the two compounds (**3b** and **3g**) showed more potent anticancer activities against HepG2 and MCF-7 than 5-FU. An MTT assay and flow cytometry showed that only **3b** and **3g** had anticancer activity and antiproliferative activities at the G1 phase against MCF-7 cells, while six compounds (**3a**-**e** and **3g**) had cytotoxicity and cell cycle arrest at different phases against HepG2 cells. Their cytotoxicity was achieved through downregulation of Bcl-2 and upregulation of Bax, caspase-3, and caspase-9. Although all tested compounds increased oxidative stress *via* increment of MDA levels and decrement of glutathione reductase (GR) activities compared to control, the **3a**, **3b**, and **3g** in HepG2 and **3b** and **3g** in MCF-7 achieved the target results. Moreover, there was a positive correlation between cytotoxic efficacy of the compound and apoptosis in both HepG2 (*R*^2^ = 0.531; *P* = 0.001) and MCF-7 (*R*^2^ = 0.219; *P* = 0.349) cell lines. The results of molecular docking analysis of **3a-g** into the binding groove of Bcl-2 revealed relatively moderate binding free energies compared to the selective Bcl-2 inhibitor, DRO. Like venetoclax, compounds **3a-g** showed 2 violations from Lipinski's rule. However, the results of the ADME study also revealed higher drug-likeness scores for compounds **3a-g** than for venetoclax. In conclusion, the tested newly synthesized chalcone-pyridothienopyrimidinone derivatives showed promising antiproliferative and apoptotic effects. Mechanistically, the compounds increased ROS production with concomitant cell cycle arrest and apoptosis. Therefore, regulation of the cell cycle and apoptosis are possible targets for anticancer therapy. The tested compounds could be potent anticancer agents to be tested in future clinical trials after extensive pharmacodynamic, pharmacokinetic, and toxicity profile investigations.

## 1. Introduction

Cancer is a multifactorial disease that results from the mutation of certain genes that regulate cell function upon exposure to specific environmental factors. It is characterized by out-of-control cell growth leading to expansive masses of abnormal cells that infiltrate and damage nearby normal tissues. According to the World Health Organization [1], cancer is the second leading cause of death globally and accounted for 7.4 million deaths in 2004 and will continue to increase with an expected 11.5 million deaths in 2030. Although significant progress has been made in cancer treatment, adverse side effects and drug resistance remain serious problems. Therefore, the search is still on for safe and effective chemotherapeutic agents for cancer treatment.

Apoptosis is a physiological process that involves programmed cell death to help the body eliminate aging and nonfunctioning cells *via* the autodigestion process [2]. Apoptosis has a critical role in cancer therapy. Any disturbance in the apoptotic pathway results in many diseases, including cancer. Therefore, the design of a novel anticancer agent that can recover the normal apoptotic pathways is a promising strategy for cancer treatment [3]. Apoptosis in tumor cells can be triggered by various natural and synthetic agents [4]. Synthetic products are frequently more potent than their original compounds [4]. Combinations of chalcones with other pharmacologically interesting scaffolds increase their advantage as anticancer therapy. Previous studies highlighted the ability of chalcones to enhance apoptosis [5].

The heterocycles of thienopyrimidine moiety are structural analogs of the natural pyrimidines “cytosine, thymine, and uracil” [6]. They consist of a thiophene ring bonded with the pyrimidine ring. Their thiophene moiety is identical to the imidazole ring in the purine ring [7]. 2-Thiouracil (a natural equivalent of thiopyrimidines) is a minor component of t-RNA. Their sulfur- or/and nitrogen-disubstituted components showed anticancer effects due to their incorporation into DNA and consequently act as effective inhibitors of nucleic acid syntheses [8]. Thienopyrimidine moiety is a constitutional part of several biologically active compounds [9]. The anticancer effects of thienopyrimidines were recently studied *via* suppression of the protein kinase family [10] and STAT protein family [11]. Due to their interesting biological activities, we have focused our interests on the synthesis of a large number of thienopyrimidine derivatives to find compounds endowed with anticancer activity.

Chalcones or 1,3-diaryl-2-propen-1-ones are an essential class of natural flavonoids and isoflavonoids that are found in many nutritional materials such as vegetables, tea, spices, and fruits. Chalcones have antibacterial [12], antifungal [13], anti-inflammatory [14], and antioxidant [15] activities. The anticancer and antiproliferative activities of chalcones have also been intensively studied against different cancer cell lines [16]. Their biological activities are due to their chemical structure and *α*,*β*-unsaturated carbonyl derivatives [17]. The most striking is that chalcones do not induce undesirable genotoxic effects as done with many useful anticancer drugs that may interact with the amino groups of purine and pyrimidine nucleotides of nucleic acids [18].

Several chemotherapeutic drugs have been demonstrated to cause the production of ROS, which aids in cancer cell death [19]. Oxidative stress is induced in HCT116, OV2008, and A2780 cells by thieno[2,3d]pyrimidine derivatives [20]. Furthermore, chalcone derivatives may contribute to the apoptosis of HepG2 cells through increased generation of ROS that ultimately alters the mitochondrial membrane potential of HepG2 cells [21]. In addition, chalcone analogs with a thieno[2,3-d]pyrimidin-2-yl group were found to cause mitochondrial membrane potential depolarization, accelerate ROS generation in HCT-116 cells, and increase the percentage of early and late apoptotic cells [22].

High levels of ROS, such as hydrogen peroxide, superoxide, and hydroxyl radicals, have been shown to cause cell cycle arrest, apoptosis, and irreversible cell damage in cancer cells [23]. Besides, excessive quantities of ROS oxidize and nitrate macromolecules such as lipids, proteins, and DNA, resulting in significant cellular damage [24].

As a result, it is critical to design and manufacture a number of chalcone-thienopyrimidine derivatives, as well as to investigate their mechanisms of promoting apoptosis in the tumor cell.

**Figure 1:**
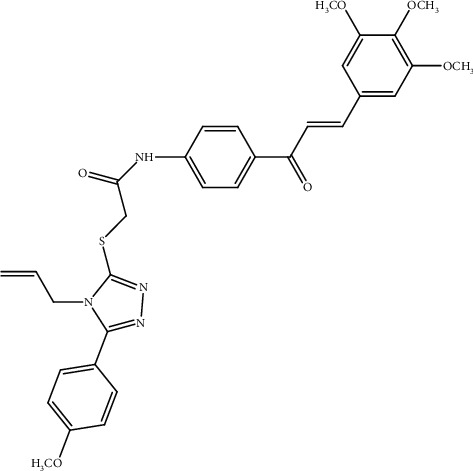
Compound I.

Over the last three decades, many hybrid molecules have been of great importance in the development of new drugs and have undergone clinical trials for the treatment of various diseases [25]. A hybridization strategy has been used to develop new anticancer drugs by fusing more than two or more active pharmacophores in a single hybrid molecule with synergistic anticancer activity [26]. Hybrid molecules are designed to enhance the biological spectrum and efficacy, overcome drug cross resistance, and reduce potential toxicity compared to the parent drugs. For example, hybridization of the 1,2,4-triazole ring with chalcone moiety in compound I (Figure 1) exhibited significant growth inhibition and induced caspase-3-dependent apoptosis in A549 human lung adenocarcinoma cells with IC_50_ value of 4.4 *μ*M relative to cisplatin with IC_50_ value of 15.3 *μ*M [27].

**Scheme 1:**
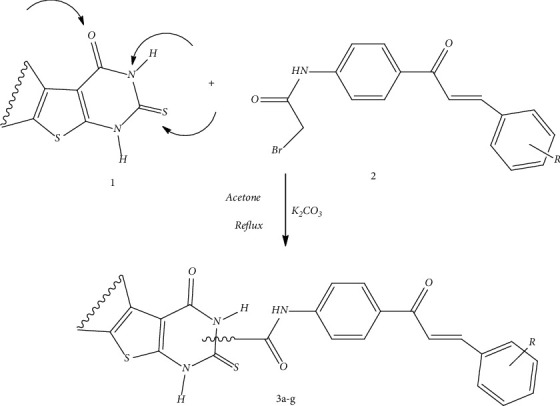
Synthesis of chalcone-thienopyrimidines **3a-g**.

Following the previous study on the importance of hybrid molecules in the treatment of different types of cancer and pathways, herein, we report the design and synthesis of certain novel thienopyrimidine/chalcone derivatives (**3a-g**, Scheme 1) that incorporate thienopyrimidine and chalcone moieties into a single compact structure for synergistic anticancer activity, manage drug resistance development, and reduce possible side effects. The synthesized compounds have different substitutions for the electron-donating and the electron-withdrawing groups for the SAR study of these compounds. The synthesized derivatives were evaluated for the cytotoxic assay and in vitro antiproliferative activity against HepG2 and MCF-7 cancer cell lines. Furthermore, the synthesized compounds were subjected to the MTT assay, cell cycle analysis, and cellular DNA content measurement. Apoptosis was measured using the Annexin V-FITC/PI apoptotic assay. Additionally, the evaluation of the relative expression levels of Bax, Bcl-2, caspase-3, and caspase-9 was measured. Finally, compounds **3a-g** were also docked into the active site of Bcl-2 to investigate the binding interaction of these compounds with different amino acids.

## 2. Materials and Methods

### 2.1. Chemicals

All used chemicals were obtained from Alfa Aesar and Fluka corporations and were used without further refinement. 5-Flurouracil was used as a standard reference anticancer agent (fluorouracil 500 mg/10 ml intravenous solution, manufactured by APP Pharmaceuticals Inc., 3 Corporate Dr, Lake Zurich, IL 60047, USA). The other used chemicals were of high purity grade and were obtained from the Aldrich Chemical Company, USA.

### 2.2. Cell Cultures

The human liver cancer cell line (HepG2 cells) and human breast cancer cell line (MCF-7 cells) were obtained from cell culture lab., Vacsera company, Egypt.

### 2.3. Synthesis of Chalcone-Thienopyrimidine Derivatives **3a-g**

The melting points of the compounds (**3a-g**) were estimated with a Gallenkamp device. Chalcone **2** (3 mmol) was mixed with a suspension of thienopyrimidine **1** (3 mmol) and K_2_CO_3_ (0.82 g, 6 mmol) in acetone (15 ml). The reaction mixture was stirred at reflux temperature for 4-6 hours. Then, the solvent was vaporized to dryness in a vacuum. The residue was diluted with water and then extracted with CH_2_Cl_2_ (3 × 30 ml). The combined organic extracts were dried over anhydrous Na_2_SO_4_, filtered, and evaporated under reduced pressure to obtain the corresponding colorless crystal product.

The synthesis of chalcone-thienopyrimidine derivatives (**3a-g**) was done according to the general pathway charted in Scheme 1. Primarily, the chalcones were created by Claisen–Schmidt condensation reaction of aromatic aldehydes with 4-methoxy acetophenone (1 : 1), using 40% potassium hydroxide as a base in ethanol. Then, compound **2** reacted with different pyridothienopyrimidine derivatives in the presence of K_2_CO_3_ under reflux in acetone to give the corresponding chalcone conjugates in 79-89% yield.

### 2.4. Identification of Compounds **3a-g**

The reactions and compounds purity were monitored by TLC, on aluminum plates coated with silica gel with a fluorescent indicator (Merck, 60 F254). The NMR spectra were performed on a JHA-LAA 400 WB-FT spectrometer (300 MHz for ^1^H NMR, 75 MHz for ^13^C NMR) and a Bruker Avance (400 MHz for ^1^H and 100 MHz for ^13^C) with either deuterated DMSO-d6 or CDCl_3_ as a solvent. Chemical shifts are expressed in *δ* using TMS as a reference. The mass spectra were recorded on Agilent 6240 Triple Quad LC-MS with methanol as a solvent.

### 2.5. Determination of the Cytotoxicity

All synthesized chalcone-thienopyrimidine derivatives **3a-g** were screened against two different cancer cell lines: HepG-2 cells and MCF-7. The MTT assay, cell cycle analysis, and apoptotic assays were used to identify the cytotoxic effect of these novel derivatives.

#### 2.5.1. Cell Lines and Culture Maintenance

Cancer cell lines were manipulated according to the method described by Sigounas et al. [28]. In this method, the cell lines were grown as adherent monolayers in T75 flasks with Dulbecco's modified Eagle's medium (DMEM) supplemented with 10% Fetal Bovine Serum (FBS), 2 mM L-glutamine, and 1% streptomycin/penicillin. The cells were left to grow in a CO_2_ incubator at 37°C, 5% CO_2_, and 95% humidity until their confluence.

#### 2.5.2. MTT Assay for Determination of Cell Proliferation

The 3-[4,5-dimethylthiazol-2-yl]-2,5-diphenyltetrazolium bromide (MTT) colorimetric assay was used to examine the sensitivity of cells to tested compounds (as anticancer drugs), as previously described [29]. This assay depends on the production of a purple formazan derivative from the yellow tetrazolium bromide (MTT) by mitochondrial succinate dehydrogenase in viable cells. The percentage of cell survival was calculated as follows:

(1) $$\begin{array}{c} \text{Percentage of survival}=\frac{\text{O}.\text{D}.\text{treated cells}}{\text{O}.\text{D}.\text{control cells}}\times 100. \end{array}$$

For preliminary screening of the cytotoxicity of synthesized compounds **3a-g**, cells exposed to DMSO were used as a control. Plotting the relationship between the survival score and the compound concentration and calculating the IC_50_ (the concentration required to inhibit cell viability by 50%) were performed for each test compound according to Li et al. [30] in comparison with the reference standard anticancer agent, 5-FU.

#### 2.5.3. Cell Cycle Analysis and Cellular DNA Content Measurement

Cell cycle analysis was performed to decide if the newly synthesized thienopyrimidine derivatives arrested the cell cycle and at what phase of the cell cycle, the HepG2 and MCF-7 cell lines were arrested. The experiments were performed as previously described by Tolba et al. [31]. HepG2 and MCF-7 cell lines were seeded and incubated with the tested compounds in 6-well plates. After incubation overnight, the cells were washed twice with ice-cold phosphate-buffered saline (PBS), detached by EDTA-trypsinization from the plates, harvested by centrifugation, fixed in ice-cold 70% (*v*/*v*) ethanol, and washed with phosphate-buffered saline (PBS, pH 7.2 ± 0.2). The cells were resuspended with 0.1 mg/ml RNase, stained with 40 mg/ml propidium iodide (PI), and analyzed by flow cytometry using a FACSCalibur flow cytometer (BD Biosciences, San Jose, CA). CellQuest software (Becton Dickinson) was used to determine the cell cycle distributions after incubation with 5-FU or tested compounds. Exposure of HepG2 and MCF-7 cell lines to these compounds could interfere with the normal cell cycle distribution.

#### 2.5.4. Measurement of Apoptosis Using the Annexin V-FITC/PI Apoptotic Assay

Annexin V-fluorescein isothiocyanate (FITC) and counterstaining with propidium iodide (PI) using the Annexin V-FITC/PI apoptosis detection kit (BD Biosciences, San Diego, CA) were employed to distinguish early and late apoptotic cells according to the manufacturer's directions. Annexin V conjugated with fluorescein isothiocyanate was used to quantify the loss of phosphatidylserine asymmetry in cell membranes involved in apoptosis, and propidium iodide can distinguish between early apoptosis, late apoptotic, and necrotic cells [32]. Analyses were achieved by using a FACSCalibur flow cytometer (BD Biosciences, San Jose, CA).

### 2.6. Evaluation of the Relative Expression Levels of Bax, Bcl-2, Caspase-3, and Caspase-9

We determined the effect of IC_50_ concentration of newly synthesized thienopyrimidine derivatives (**3a**-**e** and **3g** and **3b** and **3g**) on the relative expression levels of some markers of apoptosis in HepG2 and MCF-7 cell lines, respectively. These included the antiapoptotic marker Bcl-2 as well as the apoptotic markers Bax, caspase-3, and caspase-9 [33]. BIORAD iScript™ One-Step RT-PCR kit with SYBR® Green was used to assess the relative levels of expression of apoptosis markers. The measurement was carried out according to the technique of reverse transcription polymerase chain reaction (RT-PCR) using the Rotor-Gene RT-PCR system, and the method of the used kit was done based on the manufacturer's instructions. The procedure of this assay included the following:

#### 2.6.1. RNA Purification Using RNeasy Technology

Based on the manufacturer's instructions, the Qiagen RNeasy Extraction Kit (Qiagen Ltd., UK) was used to isolate mRNA from approximately 1 × 10^7^ cells according to the cell line.

#### 2.6.2. Master Mix Preparation

Mix all the following reagents to get the total volume (50 *μ*l): 2X SYBR® Green RTPCR reaction mixture (25 *μ*l), forward primer (10 mM) (1.5 *μ*l), reverse primer (10 mM) (1.5 *μ*l), nuclease-free water (11 *μ*l), RNA template (1 pg-100 ng total RNA) (10 *μ*l), and iScript reverse transcriptase (1 *μ*l) for One-Step RT-PCR. The real-time PCR sequences of the forward and reverse primers of the following genes were shown in Table 1.

The thermal cycler (Rotor-Gene) program used was as follows: one cycle for reverse transcription at 50°C for 10 minutes and one cycle for RT inactivation/Hot-start activation at 95°C for 5 minutes and then 45 cycles for qPCR (10 s at 95°C, 30 seconds for annealing at 55°C, and 30 seconds for extension at 72°C), and this is followed by the final extension (one cycle) at 72°C for 10 minutes.

### 2.7. Molecular Docking

The docking analysis was carried out using AutoDock 4.2 [34] to perform the docking study of compounds **3a-g** into the active site of Bcl-2 (pdb code: 2W3L) [35]. The crystal structure of Bcl-2 was retrieved from the Protein Data Bank (https://www.rcsb.org/structure). Preparation of ligands and protein files was carried out in accordance with the previous report [36]. In addition, the docking study was completed in accordance with the previous report [37]. Discovery Studio Visualizer was used to visualize the binding modes/interaction of the test compound [38].

### 2.8. ADME Study

The SwissADME web server (http://www.swissadme.ch/) [39] was used to calculate the physicochemical properties of compounds **3a-g**. The Molsoft web server (http://molsoft.com/mprop/) was also used to calculate the drug-likeness scores of the seven compounds.

### 2.9. Determination of Lipid Peroxidation (MDA)

MDA is described as a lipid peroxidation product. It reacts with thiobarbituric acid to produce a red substance that is absorbed at 535 nm [40].

### 2.10. Determination of Glutathione Reductase (GR) Activity

The activity of GR was determined by the decrease in absorbance caused by the oxidation of NADPH during the reduction of oxidized GSH [41].

### 2.11. Statistical Analysis

The obtained data were subjected to statistical analysis by the one-way analysis of variance (ANOVA) test followed by Duncan's multiple tests for comparison using the SPSS 10.0 (SPSS, Chicago, IL, USA) software program. The values were summarized as means and standard error of mean (SEM) for three replicates. The values were considered statistically significant if the *P* value was ^∗^*P* < 0.05 from control and ^#^*P* < 0.05 from 5FU. In addition, the correlation between cytotoxicity and apoptosis was determined by the Pearson correlations.

## 3. Results

### 3.1. Chemistry

#### 3.1.1. Synthesis of Chalcone-Thienopyrimidine Derivatives

**Figure 2:**
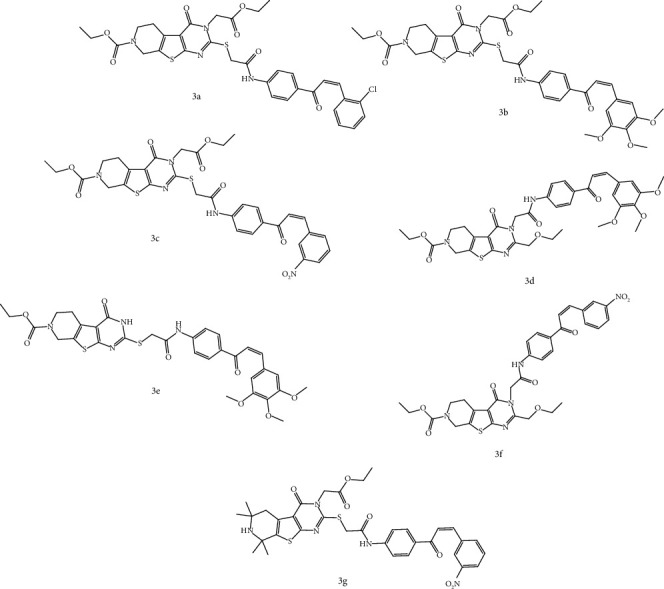
Chemical structures of synthesized chalcone-thienopyrimidines **3a-g**.

Our data indicated that in general, the synthesis of chalcone-thienopyrimidine compounds from the correspondent thienopyrimidine proceeds in a relatively short reaction time with good yield and high purity. The reaction produces only one diastereomer, which is considered *Z*-isomer, as shown in Scheme 1. The ^1^H NMR proves that there is an olefinic-H proton that is affected by the carbonyl group, since this proton appeared deshielded in the ^1^H NMR spectra of **3a-g** at *δ* > 7.60 and low coupling constant referring to the *Z*-isomer. In this study, we synthesized a series of novel chalcone-pyridothienopyrimidinone derivatives **3a-g** by reacting chalcones with different pyridothienopyrimidinone **2** according to the methods of Sauter et al. [42] and Ameen et al. [43] under reflux temperature as indicated in Figure 2.

Melting points were determined on a Boetius melting point apparatus and are uncorrected. Elemental analyses were performed on Carlo Erba CHN-S Elemental analyses 1108. The NMR spectra were obtained using a Bruker Avance (400 MHz for ^1^H and 100 MHz for ^13^C), Institute of Organic Chemistry, Karlsruhe University, Karlsruhe, Germany, with deuterated DMSO-d_6_ as a solvent. Chemical shift is quoted in *δ* and is referenced to TMS. Mass spectrometry was performed by electron impact at 70 eV (FAB-MS): Finnigan MAT 95, Institute of Organic Chemistry, Karlsruhe Institute of Technology, Karlsruhe, Germany. The reactions and purity were monitored by TLC, on aluminum plates coated with silica gel with a fluorescent indicator (Merck, 60 F254).

The IUPAC nomenclature, chemical formula, molecular weight, melting point (m.p.), and NMR (^1^H NMR and ^13^C NMR) analysis (Supplementary 3) of the synthesized chalcone-thienopyrimidine derivatives are presented as follows.

### 3.2. Compound **3a**

#### 3.2.1. Ethyl (Z)-2-((2-((4-(3-(2-Chlorophenyl)acryloyl)phenyl)amino)-2-oxoethyl)thio)-3-(2-ethoxy-2-oxoethyl)-4-oxo-3,5,6,8-tetrahydropyrido[4′,3′:4,5]thieno[2,3-*d*]pyrimidine-7(4*H*)-carboxylate

Colorless crystals from DMF-H_2_O; Yield (79%); m.p. 181-182°C. ^1^H NMR (400 MHz, *DMSO*, ppm): *δ* 1.26 (t, 6H, J =7.1 Hz, 2COOCH_2_*CH_3_*), 2.71 (t, 2H, J =5.6 Hz, H-5), 2.87 (t, 2H, J =5.1 Hz, H-6), 4.09 (q, 2H, J =7.0 Hz, COO*CH_2_*CH_3_), 4.21 (q, 2H, J =7.0 Hz, COO*CH_2_*CH_3_), 4.29 (s, 2H, SCH_2_), 4.59 (s, 2H, H-8). 4.92 (s, 2H, NCH_2_), 7.45-7.60 (m, 4H, Ar-H), 7.79 (d, 2H, J =9.0 Hz, Ar-H), 8.01 (d, 2H, J =4.0 Hz, 2CH), 8.20 (d, 2H, J =9.0 Hz, Ar-H), 10.78 (s, 1H, NH). ^13^C NMR (100 MHz, *DMSO*, ppm): *δ* 14.4 (CH_3_), 14.7 (CH_3_), 30.1 (C-5), 35.7 (SCH_2_), 40.7 (C-6), 42.8 (C-8), 44.7 (NCH_2_), 61.1 (CH_2_, carbamate), 61.6 (CH_2_, carbamate), 117.1 (C-4a), 124.6 (C-4b), 127.6 (C-8a), 128.4, 129.2, 129.9, 130.1, 131.8, 132.2, 132.4, 134.3, 137.9, 141.3 (12 C-Ar, 2 CH-olefine), 154.6 (C-9a), 154.9, 156.8, 161.7, 162.3, 185.3 (5 C=O), (EI-MS): *m/z* calcd. C_33_H_31_ClN_4_O_7_S_2_ [M]^+^: 694.13, found: 694.11. Anal. Calcd for C_33_H_31_ClN_4_O_7_S_2_ (695.20): C, 57.01; H, 4.49; Cl, 5.10; N, 8.06; Found: C, 56.96; H, 4.38; Cl, 5.02; N, 7.98.

### 3.3. Compound **3b**

#### 3.3.1. Ethyl (Z)-3-(2-Ethoxy-2-oxoethyl)-4-oxo-2-((2-oxo-2-((4-(3-(3,4,5 trimethoxyphenyl) acryloyl)phenyl)amino)ethyl)thio)-3,5,6,8 tetra-hydropyrido[4′,3′:4,5]thieno[2,3-*d*]pyrimidine-7(4*H*)-carboxylate

Colorless crystals from DMF-H_2_O; Yield (81%); m.p. 172-173°C. ^1^H NMR (400 MHz, *DMSO*, ppm): *δ* 1.11, 1.22 (t, 6H, J =7.1 Hz, 2COOCH_2_*CH_3_*), 2.86 (t, 2H, J =5.6 Hz, H-5), 3.61 (t, 2H, J =5.1 Hz, H-6), 3.86 (s, 3H, OCH_3_), 3.97 (s, 6H, 2OCH_3_), 4.08 (q, 2H, J =7.0 Hz, COO*CH_2_*CH_3_), 4.20 (q, 2H, J =7.0 Hz, COO*CH_2_*CH_3_), 4.26 (s, 2H, SCH_2_), 4.55 (s, 2H, H-8). 4.87 (s, 2H, NCH_2_), 7.65-7.95 (m, 2H, Ar-H, d, 2H, J =9.0 Hz, Ar-H, d, 2H, J =4.0 Hz, 2CH), 8.20 (d, 2H, J =9.0 Hz, Ar-H), 10.77 (s, 1H, NH). ^13^C NMR (100 MHz, *DMSO*, ppm): *δ* 13.9 (CH_3_), 14.5 (CH_3_), 30.1 (C-5), 35.5 (SCH_2_), 39.7 (C-6), 42.5 (C-8), 44.5 (NCH_2_), 56.1 (2OCH_3_), 59.2 (OCH_3_), 61.1 (CH_2_, carbamate), 61.6 (CH_2_, carbamate), 105.6 (2CH-Ar), 117.1 (C-4a), 120.1 (CH-Ar), 122.2, (CH-olefine), 124.6 (C-4b), 127.6, 128.8 (C-8a), 129.9, 130.1, 131.8, 132.2, 132.6, 137.9, 139.5 (6 C-Ar, CH-Ar), 143.2 (CH-olefine), 152.6 (C-9a), 155.6, 156.7, 162.6, 165.3, 186.6 (5 C=O), (EI-MS): *m/z* calcd. C_36_H_38_N_4_O_10_S_2_ [M]^+^: 750.20, found: 750.23. Anal. Calcd for C_36_H_38_N_4_O_10_S_2_ (750.84): C, 57.59; H, 5.10; N, 7.46; Found: C, 57.47; H, 5.01; N, 7.36.

### 3.4. Compound **3c**

#### 3.4.1. Ethyl (Z)-3-(2-Ethoxy-2-oxoethyl)-2-((2-((4-(3-(3-nitrophenyl)acryloyl)phenyl)amino)-2-oxoethyl)thio)-4-oxo-3,5,6,8-tetrahydropyrido[4′,3′:4,5]thieno[2,3-*d*]pyrimidine-7(4*H*)-carboxylate

Pale yellow crystals from DMF-H_2_O; Yield (86%); m.p. 189-190°C. ^1^H NMR (400 MHz, *DMSO*, ppm): *δ* 1.14 (t, 3H, J =7.1 Hz, COOCH_2_*CH_3_*), 1.21 (t, 3H, J =7.1 Hz, COOCH_2_*CH_3_*), 2.78 (t, 2H, J =5.6 Hz, H-5), 3.64 (t, 2H, J =5.1 Hz, H-6), 4.11 (q, 2H, J =7.0 Hz, COO*CH_2_*CH_3_), 4.21 (q, 2H, J =7.0 Hz, COO*CH_2_*CH_3_), 4.31 (s, 2H, SCH_2_), 4.58 (s, 2H, H-8). 4.95 (s, 2H, NCH_2_), 7.51-7.68 (m, 4H, Ar-H), 7.79 (d, 2H, J =9.0 Hz, Ar-H), 8.12 (d, 2H, J =4.0 Hz, 2CH), 8.20 (d, 2H, J =9.0 Hz, Ar-H), 10.82 (s, 1H, NH). ^13^C NMR (100 MHz, *DMSO*, ppm): *δ* 14.0 (CH_3_), 14.7 (CH_3_), 31.1 (C-5), 35.6 (SCH_2_), 40.8 (C-6), 42.9 (C-8), 44.7 (NCH_2_), 61.2 (CH_2_, carbamate), 61.6 (CH_2_, carbamate), 118.1 (C-4a), 125.6 (C-4b), 127.6 (C-8a), 128.4, 129.3, 129.9, 130.2, 131.9, 132.4, 132.6, 134.5, 137.9, 141.4 (12 C-Ar, 2 CH-olefine), 154.6 (C-9a), 155.6, 156.8, 162.6, 165.6, 185.4 (5 C=O), (EI-MS): *m/z* calcd. C_33_H_31_N_5_O_9_S_2_ [M]^+^: 705.16, found: 705.21. Anal. Calcd for C_33_H_31_N_5_O_9_S_2_ (705.76): C, 56.16; H, 4.43; N, 9.92; Found: C, 56.04; H, 4.32; N, 9.81.

### 3.5. Compound **3d**

#### 3.5.1. Ethyl (Z)-2-(Ethoxymethyl)-4-oxo-3-(2-oxo-2-((4-(3-(2,3,4-trimethoxyphenyl)acryloyl)-phenyl)amino)ethyl)-3,5,6,8-tetrahydropyrido[4′,3′:4,5]thieno[2,3-*d*]pyrimidine-7(4*H*)-carboxylate

Pale yellow crystals from DMF-H_2_O; Yield (80%); m.p. 157-158°C. ^1^H NMR (400 MHz, *DMSO*, ppm): *δ* 1.07 (t, 3H, J =7.1 Hz, OCH_2_*CH_3_*), 1.22 (t, 3H, J =7.1 Hz, COOCH_2_*CH_3_*), 2.89 (t, 2H, J =5.6 Hz, H-5), 3.48 (q, 2H, J =7.0 Hz, O*CH_2_*CH_3_), 3.65 (t, 2H, J =5.1 Hz, H-6), 3.76 (s, 3H, OCH_3_), 3.87 (s, 6H, 2OCH_3_), 4.10 (q, 2H, J =7.0 Hz, OCH_2_*CH_3_*), 4.20 (q, 2H, J =7.0 Hz, COO*CH_2_*CH_3_), 4.51 (s, 2H, OCH_2_), 4.59 (s, 2H, H-8). 4.98 (s, 2H, NCH_2_), 7.14 (d, 2H, J =9.0 Hz, Ar-H), 7.71-7.96 (m, 2H, Ar-H, d, 2H, J =4.0 Hz, 2CH), 8.20 (d, 2H, J =9.0 Hz, Ar-H), 10.79 (s, 1H, NH). ^13^C NMR (100 MHz, *DMSO*, ppm): *δ* 14.5 (CH_3_), 14.7 (CH_3_), 39.5 (C-5), 35.7 (OCH_2_), 39.9 (C-6), 42.5 (C-8), 55.5 (NCH_2_), 56.1 (2OCH_3_), 60.1 (OCH_3_), 61.1 (CH_2_, O*CH_2_*CH_3_), 65.6 (CH_2_, carbamate), 110.6 (2CH-Ar), 111.2 (C-4a), 121.1, 122.3 (4 CH-Ar), 124.6 (C-4b), 127.6, (C-8a), 132.2, 133.6, 137.9, 138.5 (C-Ar), 143.2 (2 CH-olefine), 150.1 (C-9a), 157.6, 159.1 (3 *C*-OCH_3_), 162.6, 162.3, 171.2, 191.4 (4 C=O), (EI-MS): *m/z* calcd. C_35_H_38_N_4_O_9_S [M]^+^: 690.24, found: 690.16. Anal. Calcd for C_35_H_38_N_4_O_9_S (690.77): C, 60.86; H, 5.55; N, 8.11; Found: C, 60.77; H, 5.43; N, 8.04.

### 3.6. Compound **3e**

#### 3.6.1. Ethyl (Z)-4-Oxo-2-((2-oxo-2-((4-(3-(3,4,5-trimethoxyphenyl)acryloyl)phenyl)amino)ethyl)-thio)-3,5,6,8-tetrahydropyrido[4′,3′:4,5]thieno[2,3-*d*]pyrimidine-7(4*H*)-carboxylate

Pale yellow crystals from DMF-H_2_O; Yield (89%); m.p. 167-168°C. ^1^H NMR (400 MHz, *DMSO*, ppm): *δ* 1.18 (t, 3H, J =7.1 Hz, COOCH_2_*CH_3_*), 2.86 (t, 2H, J =5.6 Hz, H-5), 3.64 (t, 2H, J =5.1 Hz, H-6), 3.86 (s, 3H, OCH_3_), 3.88 (s, 6H, 2OCH_3_), 4.22 (q, 2H, J =7.0 Hz, COO*CH_2_*CH_3_), 4.27 (s, 2H, SCH_2_), 4.58 (s, 2H, H-8), 7.55-7.95 (m, 2H, Ar-H, d, 2H, J =9.0 Hz, Ar-H, d, 2H, J =4.0 Hz, 2CH), 8.20 (d, 2H, J =9.0 Hz, Ar-H), 10.77 (s, 1H, NH), 11.17 (s, 1H, NH). ^13^C NMR (100 MHz, *DMSO*, ppm): *δ* 14.5 (CH_3_), 30.1 (C-5), 35.5 (SCH_2_), 39.7 (C-6), 42.5 (C-8), 56.1 (2OCH_3_), 59.2 (OCH_3_), 61.6 (CH_2_, carbamate), 107.6 (2CH-Ar), 117.4 (C-4a), 120.5 (CH-Ar), 121.2, (CH-olefine), 123.6 (C-4b), 128.6, 128.8 (C-8a), 129.7, 130.1, 130.8, 132.1, 132.7, 137.9, 139.5 (6 C-Ar, CH-Ar), 143.3 (CH-olefine), 152.9 (C-9a), 153.6, 156.5, 164.3, 186.7 (4 C=O), (EI-MS): *m/z* calcd. C_32_H_32_N_4_O_8_S_2_ [M]^+^: 664.17, found: 664.25. Anal. Calcd for C_32_H_32_N_4_O_8_S_2_ (664.75): C, 57.82; H, 4.85; N, 8.43; Found:): C, 57.71; H, 4.75; N, 8.32.

### 3.7. Compound **3f**

#### 3.7.1. Ethyl (Z)-2-(Ethoxymethyl)-3-(2-((4-(3-(3-nitrophenyl)acryloyl)phenyl)amino)-2-oxoethyl)-4-oxo-3,5,6,8-tetrahydropyrido[4′,3′:4,5]thieno[2,3-*d*]pyrimidine-7(4*H*)-carboxylate

Pale yellow crystals from DMF-H_2_O; Yield (82%); m.p. 178-179°C. ^1^H NMR (400 MHz, *DMSO*, ppm): *δ* 1.14 (t, 3H, J =7.1 Hz, OCH_2_*CH_3_*), 1.21 (t, 3H, J =7.1 Hz, COOCH_2_*CH_3_*), 2.78 (t, 2H, J =5.6 Hz, H-5), 3.64 (t, 2H, J =5.1 Hz, H-6), 4.11 (q, 2H, J =7.0 Hz, O*CH_2_*CH_3_), 4.21 (q, 2H, J =7.0 Hz, COO*CH_2_*CH_3_), 4.31 (s, 2H, CH_2_O), 4.58 (s, 2H, H-8). 4.95 (s, 2H, NCH_2_), 7.51-7.68 (m, 4H, Ar-H), 7.79 (d, 2H, J =9.0 Hz, Ar-H), 8.12 (d, 2H, J =4.0 Hz, 2CH), 8.20 (d, 2H, J =9.0 Hz, Ar-H), 10.82 (s, 1H, NH). ^13^C NMR (100 MHz, *DMSO*, ppm): *δ* 14.1 (CH_3_), 14.6 (CH_3_), 31.4 (C-5), 35.8 (OCH_2_), 41.5 (C-6), 42.7 (C-8), 44.8 (NCH_2_), 61.2 (CH_2_, O*CH_2_*CH_3_), 61.6 (CH_2_, carbamate), 117.9 (C-4a), 124.6 (C-4b), 127.6 (C-8a), 128.3, 129.5, 129.9, 130.6, 131.2, 131.8, 132.4, 132.6, 134.5, 137.9, 141.4 (12 C-Ar, 2 CH-olefine), 154.6 (C-9a), 155.7, 156.7, 166.5, 184.4 (4 C=O), (EI-MS): *m/z* calcd. C_32_H_31_N_5_O_8_S [M]^+^: 645.19, found: 645.21. Anal. Calcd for C_32_H_31_N_5_O_8_S (645.69); C, 59.53; H, 4.84; N, 10.85; Found: C, 59.42; H, 4.73; N, 10.75.

### 3.8. Compound **3g**

#### 3.8.1. Ethyl (Z)-2-(6,6,8,8-Tetramethyl-2-((2-((4-(3-(3-nitrophenyl)acryloyl)-phenyl)amino)-2-oxoethyl)thio)-4-oxo-5,6,7,8-tetrahydropyrido[4′,3′:4,5]thieno[2,3-*d*]pyrimidin-3(4*H*)-yl)acetate

Pale yellow crystals from DMF-H_2_O; Yield (80%); m.p. 194-195°C. ^1^H NMR (400 MHz, *DMSO*, ppm): *δ* 1.13 (t, 3H, J =7.1 Hz, COOCH_2_*CH_3_*), 1.23 (s, 6H, 2CH_3_), 1.23 (s, 6H, 2CH_3_), 2.03 (s, H, NH), 2.76 (t, 2H, J =5.6 Hz, H-5), 4.12 (q, 2H, J =7.0 Hz, COO*CH_2_*CH_3_), 4.33 (s, 2H, SCH_2_), 4.88 (s, 2H, NCH_2_), 7.67-7.65 (m, 4H, Ar-H), 7.76 (d, 2H, J =9.0 Hz, Ar-H), 8.09 (d, 2H, J =4.0 Hz, 2CH), 8.21 (d, 2H, J =9.0 Hz, Ar-H), 10.67 (s, 1H, NH). ^13^C NMR (100 MHz, *DMSO*, ppm): *δ* 14.1 (CH_3_), 28.1 (2CH_3_), 33.2 (C-5), 34.1 (2CH_3_), 35.7 (SCH_2_), 44.6 (NCH_2_), 58.9 (C-6), 61.1 (C-8), 61.3 (CH_2_, carbamate), 117.2 (C-4a), 124.6 (C-4b), 127.7 (C-8a), 128.3, 129.3, 130.1, 131.2, 131.8, 132.6, 132.5, 134.8, 136.9, 141.6 (12 C-Ar, 2 CH-olefine), 153.6 (C-9a), 155.7, 156.4, 161.8, 184.3 (4 C=O), (EI-MS): *m/z* calcd. C_34_H_35_N_5_O_7_S_2_ [M]^+^: 689.20, found: 689.27. Anal. Calcd for C_34_H_35_N_5_O_7_S_2_ (689.80); C, 59.20; H, 5.11; N, 10.15; Found: C, 59.04; H, 5.07; N, 10.08.

### 3.9. Cytotoxic Activity (MTT Assay)

The results presented in Tables 2 and 3 indicate the percentage of survival of the two cell lines (HepG2 and MCF-7) after exposure to serial dilution of the synthesized chalcone-thienopyrimidine derivatives **3a-g** as well as DMSO.

DMSO (at dilution rate 1.5625) showed no cytotoxicity on MCF-7 and HepG2 cell lines producing survival percentages of 102.6 ± 0.632 and 103.6 ± 0.051, respectively. Therefore, we suggested that the tested compounds showing cell survival percentage less than 100% at the corresponding dilution had cytotoxic efficacy and then they were used in further experiments.

**Figure 3:**
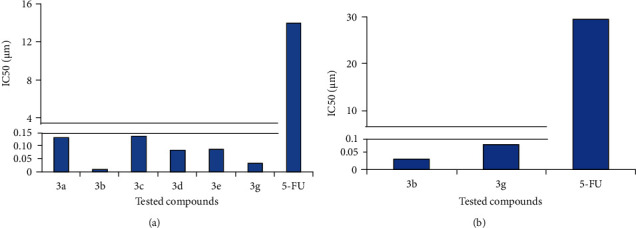
Cytotoxicity for some newly synthesized thienopyrimidine derivatives **3a-g** against (a) HepG2 and (b) MCF-7 cancer cell lines in comparison to the traditional anticancer drug 5-FU. IC_50_ of triplicates was expressed as *μ*M.

The MTT assay results revealed that the six compounds **3a**, **3b**, **3c**, **3d**, **3e**, and **3g** exhibited potent cytotoxicity with IC_50_ values ranging from 0.0332 ± 0.0028 to 0.1321 ± 0.0152 *μ*M against the HepG2 cell line. Moreover, the two compounds **3b** and **3g** showed the maximum anticancer effects with the lowest IC_50_ values of 0.0073 ± 0.0016 and 0.0332 ± 0.0028 *μ*M, respectively. The two compounds **3b** and **3g** showed anticancer effects which represented 4046.90- and 420.94-fold more potent activity than 5-FU (13.9753 ± 0.149 *μ*M) (Figure 3(a)).

The MTT assay results of the tested compounds **3a**, **3b**, and **3c** on the HepG2 cell line indicate that compound **3b** containing 3-methoxy-donating function groups on chalcone moiety exhibited remarkable anticancer activity (~18 times more activity) compared to compounds **3a** and **3c** containing an electron-withdrawing function group on chalcone moiety.

Compounds **3d** and **3e** containing 3-methoxy-donating function groups on chalcone moiety either on the sulfur atom or on the nitrogen atom of thienopyrimidine exhibited nearly equal anticancer activity with IC_50_ of 0.0837 *μ*M and 0.0871 *μ*M, respectively.

Compound **3g** containing an electron-withdrawing function group (NO_2_) with the absence of ethoxycarbonyl moiety on the nitrogen of piperidine moiety exhibited good anticancer activity with IC_50_ of 0.0033 *μ*M.

On the other hand, only **3b** and **3g** compounds were the most potent cytotoxic compounds with IC_50_ values of 0.0349 ± 0.0047 and 0.0843 ± 0.0066 *μ*M, respectively, representing 846.5- and 350.4-fold more effective anticancer activity than 5-FU (29.5424 ± 0.264 *μ*M) against the MCF-7 cell line (Figure 3(b)).

The MTT assay results of the tested compounds **3b** and **3g** on the MCF-7 cell line indicate that compound **3b** containing 3-methoxy-donating function groups on chalcone moiety exhibited remarkable anticancer activity (~2.5 times more activity) compared to compound **3g** containing an electron-withdrawing function group (NO_2_) on chalcone moiety.

The most active newly synthesized compounds were selected for further evaluation.

### 3.10. Cell Cycle Analysis

**Figure 4:**
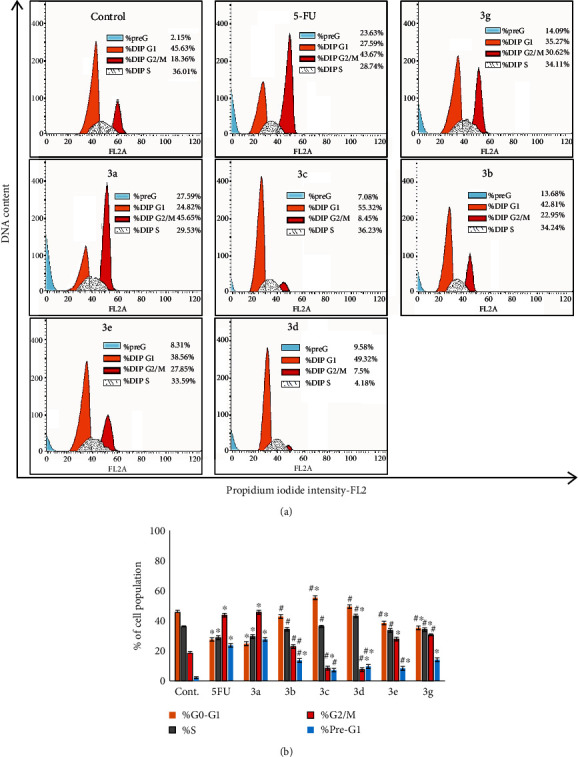
Effect of six tested compounds (**3g**, **3a**, **3c**, **3b**, **3d**, and **3e**) on cell proliferation and cell cycle phases of HepG2. (a) Change in the cell cycle of untreated (cont.), 5-fluorouracil (5-FU), and treated HepG2 was analyzed using a flow cytometer. (b) The percentage of the cell population in the phases of the HepG2 cell cycle. Data were expressed for each bar as mean ± standard error of mean. ^∗^*P* < 0.05 from control, ^#^*P* < 0.05 from 5FU.

**Figure 5:**
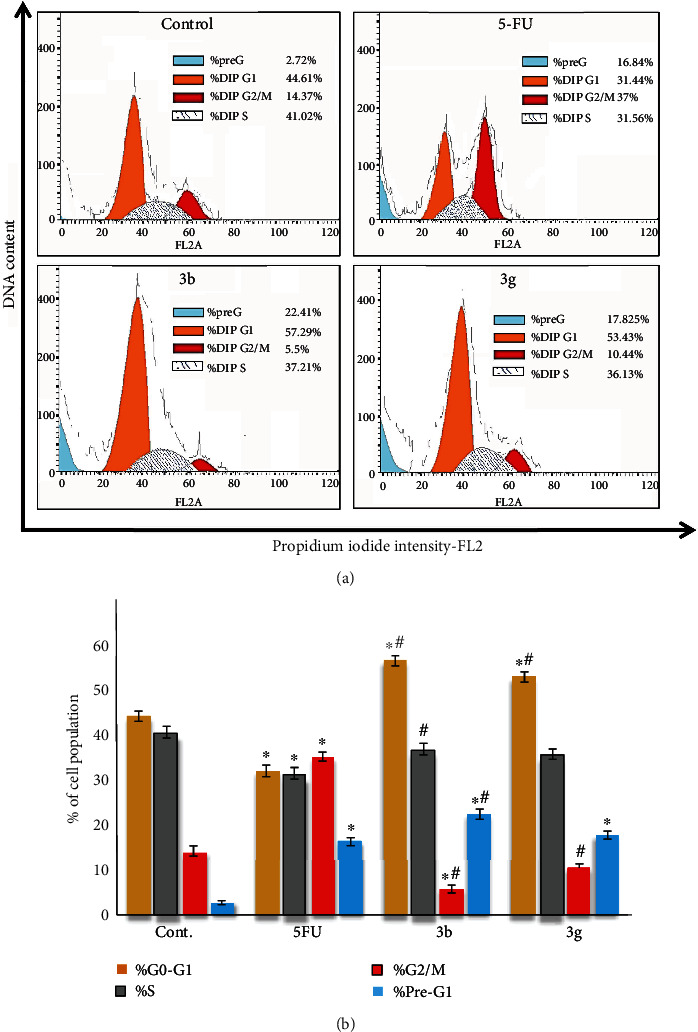
Effect of two tested compounds (**3b** and **3g**) on cell proliferation and cell cycle phases of MCF-7. (a) Change in the cell cycle of untreated (cont.), 5-fluorouracil (5-FU), and treated MCF-7 was analyzed using a flow cytometer. (b) The percentage of the cell population in the phases of the MCF-7 cell cycle. Data were expressed for each bar as mean ± standard error of mean. ^∗^*P* < 0.05 from control, ^#^*P* < 0.05 from 5FU.

The molecular mechanism of antiproliferation induction of the HepG2 cell line by the six tested compounds **3a**, **3b**, **3c**, **3d**, **3e**, and **3g** as well as the MCF-7 cell line by two compounds **3b** and **3g** was evaluated by measuring their impacts on cell cycle progression using propidium iodide. The data presented in Figures 4 and 5 illustrated the flow cytometry analyses of cell cycle distribution in the untreated cell (control), 5-fluorouracil (5-FU), and tested newly synthesized chalcone-thienopyrimidine derivatives.

Flow cytometry analysis displayed that the treatment of the HepG2 cell line with compounds **3a**, **3e**, and **3g** showed substantial increase in the cell population in G2/M while compounds **3a**, **3b**, **3d**, **3e**, and **3g** induced a significant increase in pre-G1 phases compared with control cells (Figure 4). Interestingly, the application of compound **3c** exhibited a significant increase in the G1 phase cell population (*P* < 0.05). Worthwhile, compound **3c** induced a significant (*P* < 0.05) increase in the cell population in the G1 phase up to 55.32%. Additionally, compound **3d** induced a significant (*P* < 0.05) increase in the S phase cell population (Figure 4).

Regarding the MCF-7 cell line, our results revealed that compounds **3b** and **3g** significantly (*P* < 0.05) induced G1 and pre-G1 arrest. Results indicated the increased cell population in G1 and pre-G1 phases compared with control cells (Figure 5).

### 3.11. Induction of Apoptosis

**Figure 6:**
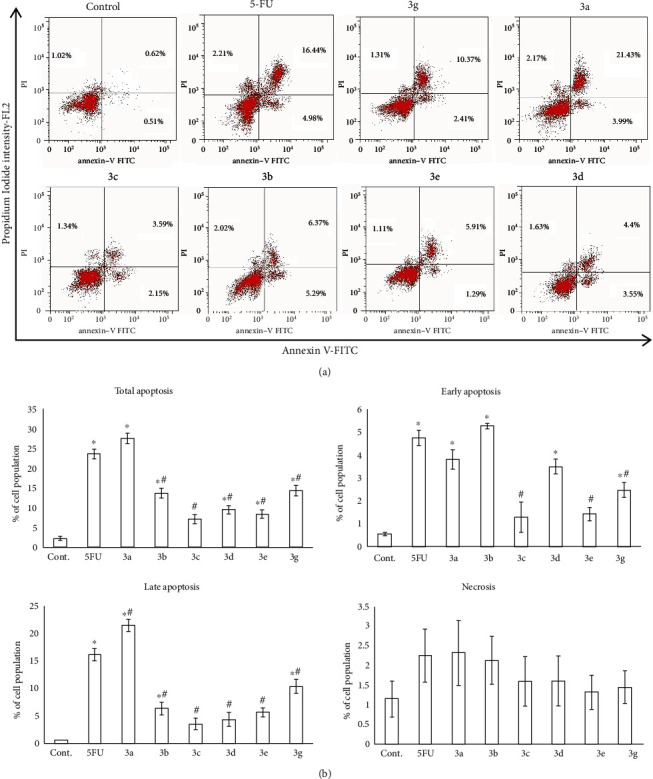
Evaluation of apoptosis in HepG2 cells treated with the six tested compounds (**3g**, **3a**, **3c**, **3b**, **3e**, and **3d**). (a) Apoptosis was analyzed using Annexin V-FITC/PI staining and flow cytometry. The right lower quadrant demonstrates early apoptotic cells, and the right upper quadrant demonstrates late apoptotic cells. (b) The bar graph showed quantification of the percentage of early and late apoptotic HepG2 cells. Data were expressed for each bar as mean ± standard error of mean. ^∗^*P* < 0.05 from control, ^#^*P* < 0.05 from 5FU.

To further demonstrate that the antiproliferative activities of the six tested compounds relied mainly on apoptosis, Annexin V-FITC/PI staining by flow cytometry (Figure 6) was used to determine the quantitative assessment of apoptosis. For HepG2 cells treated with the six compounds, the total apoptosis was evaluated; also, the cells undergoing early apoptosis (Annexin+/PI−), late apoptosis (Annexin+/PI+), and necrosis (Annexin−/PI+) were evaluated (Figure 6).

Interestingly, our results revealed that the HepG2 cell line treated with all tested compounds except **3c** showed significant induction of total apoptosis in relation to control (*P* < 0.05). On exposure to compounds **3a**, **3b**, and **3g**, the HepG2 cell line significantly exhibited early and late apoptosis (*P* < 0.05). All of these compounds elicited apoptotic effects comparable to that of 5-FU (*P* > 0.05). Moreover, no one of the tested derivatives displayed a necrotic effect in comparison with the control (*P* > 0.05) (Figure 6).

**Figure 7:**
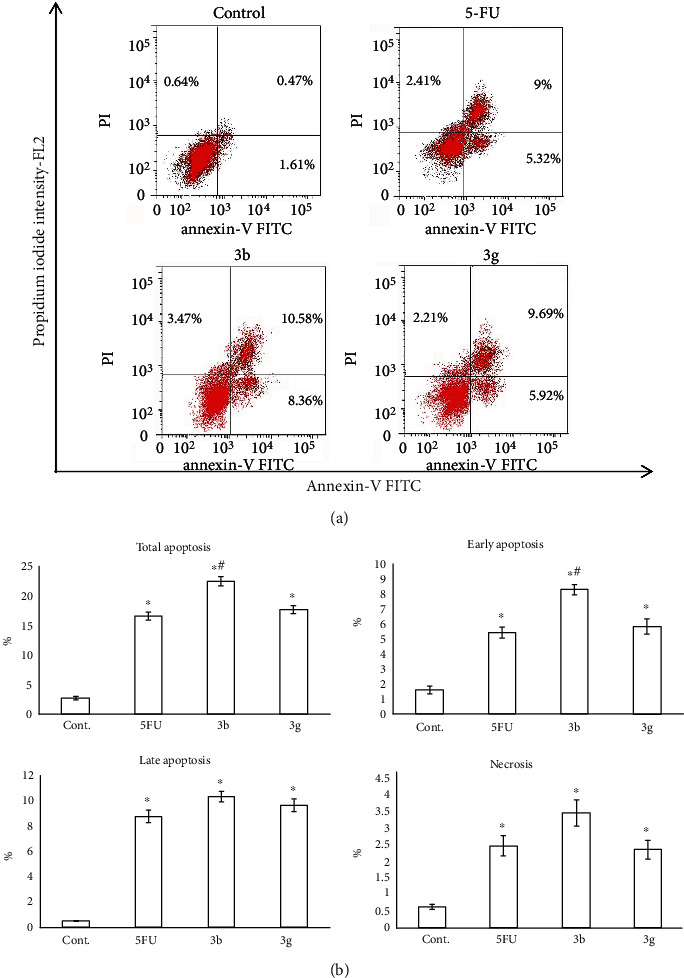
Evaluation of apoptosis in the MCF-7 cell line treated with the six tested compounds (**3b** and **3g**). (a) Apoptosis was analyzed using Annexin V-FITC/PI staining and flow cytometry. The right lower quadrant demonstrates early apoptotic cells, and the right upper quadrant demonstrates late apoptotic cells. (b) The bar graph showed quantification of the percentage of the early and late apoptotic MCF-7 cell line. Data were expressed for each bar as mean ± standard error of mean. ^∗^*P* < 0.05 from control, ^#^*P* < 0.05 from 5FU.

Concerning the MCF-7 cells, their treatment with the compounds **3a** and **3b** showed a significant increase in the total, early, and late apoptosis as well as necrosis relative to the control cells (*P* < 0.05) together with enhancing the translocation of phosphatidylserine (Annexin V-positive cells) (Figure 7).

### 3.12. Gene Expression of Bcl-2, Bax, Caspase-3, and Caspase-9

**Figure 8:**
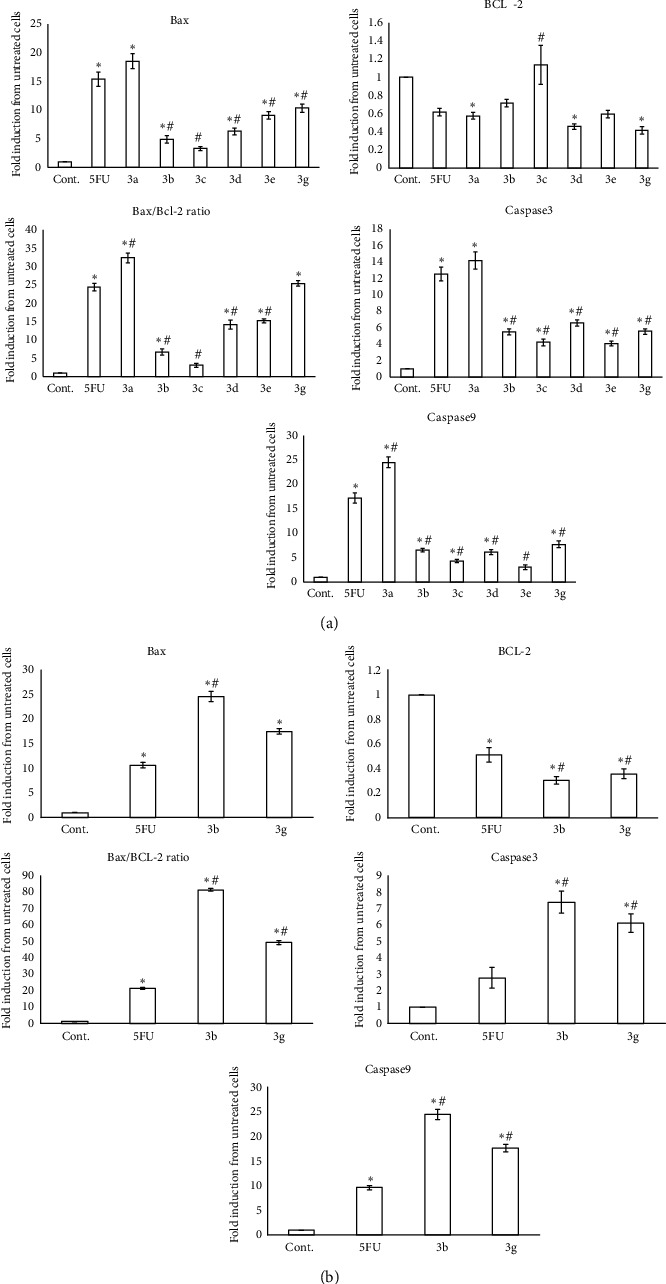
Effect of newly synthesized chalcone-thienopyrimidine derivatives **3a-e** and **3g** (IC_50_) (a) and the newly synthesized chalcone-thienopyrimidine derivatives **3b** and **3g** (IC_50_) (b) on the gene expression of Bcl-2, Bax, Bax/Bcl-2 ratio, caspase-3, and caspase-9 in HepG2 (a) and MCF-7 (b) cell lines after 8-hour incubation. Data were expressed for each bar as mean ± standard error of mean. ^∗^*P* < 0.05 from control, ^#^*P* < 0.05 from 5FU.

For further understanding of the mechanism by which the tested compounds induced apoptosis, the gene expression levels of proapoptotic factors Bax, caspase-9, and caspase-3 and antiapoptotic one Bcl-2 were measured by RT-PCR and compared to that of 5-FU (Figure 8).

For the HepG2 cell line, it is worth mentioning that all tested compounds significantly increased the expression levels of caspase-3 in comparison to control (*P* < 0.05). However, all tested compounds except **3c** revealed a marked increase in the expression levels of Bax. Furthermore, the expression levels of caspase-9 were significantly elevated with all tested compounds except **3e**. On the other side, the expression levels of Bcl-2 were noticeably decreased with **3a**, **3d**, and **3g** in comparison with untreated control cells (*P* < 0.05), while expression levels of Bcl-2 were insignificantly decreased with the other compounds (*P* > 0.05). Therefore, all tested compounds except **3c** had been shown to produce significant altitude in the Bax/Bcl-2 ratio (*P* < 0.05) in comparison to control, which supported their ability to promote the apoptotic response in HepG2 cells (Figure 8(a)).

Remarkably, in the MCF-7 cell line, the two compound chalcone-thienopyrimidine derivatives **3b** and **3g** showed significant elevation of the expression levels of the key genes of apoptosis (Bax, caspase-3, and caspase-9) and a remarkable decrease in the expression levels of Bcl-2 in relation to the control (*P* < 0.05). Therefore, Bax/Bcl-2 ratios were strikingly elevated with both **3b** and **3g** (Figure 8(b)).

### 3.13. Correlation between Cytotoxic Efficacy of the Compounds and Apoptosis

**Figure 9:**
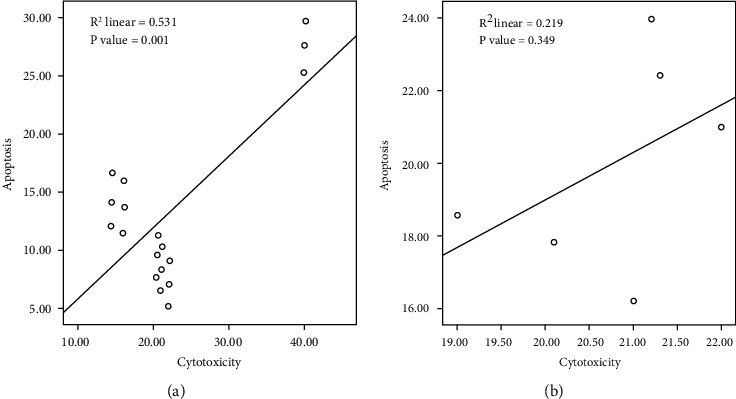
Correlation of cytotoxic efficacy of the compounds vs. apoptosis in HepG2 (a) and MCF-7 (b) cell lines.

As shown in Figure 9, there was a positive correlation between cytotoxic efficacy of the compounds and apoptosis in both HepG2 (*R*^2^ = 0.531; *P* = 0.001; Figure 9(a)) and MCF-7 (*R*^2^ = 0.219; *P* = 0.349; Figure 9(b)) cell lines.

### 3.14. Molecular Docking

**Figure 10:**
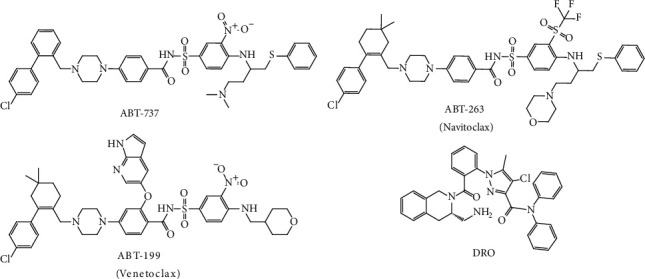
Chemical structure of Bcl-2 inhibitors.

Recently, several small molecules (1 and 2) were reported as Bcl-2 inhibitors [35, 44] (Figure 10). The ability of these molecules to inhibit the antiapoptotic Bcl-2 protein was associated with the sensitization of cancer cells to apoptosis [35]. The mechanism of action of these inhibitors was dependent on their binding to the binding groove in Bcl-2 which resulted in the inhibition of the antiapoptotic effect of Bcl-2.

**Figure 11:**
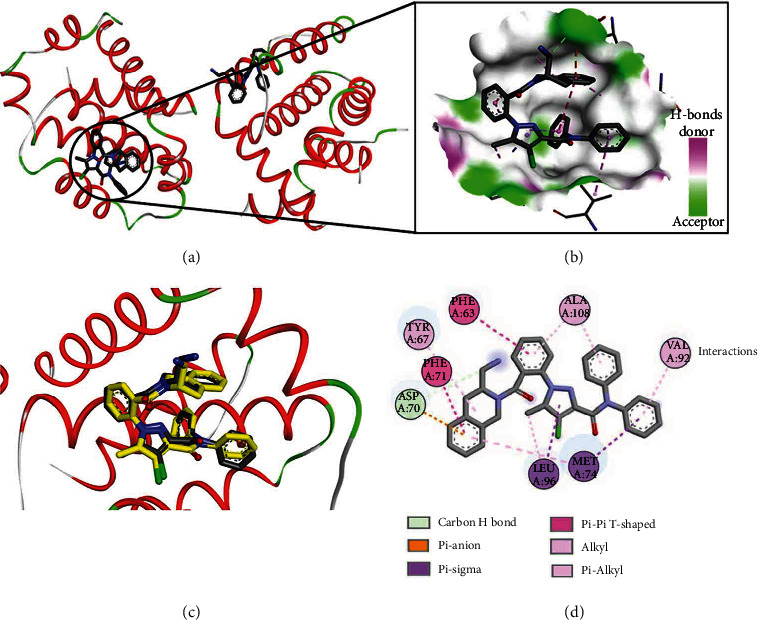
Binding mode/interactions of DRO (shown as sticks colored by element) into Bcl-2/Bcl-xL (pdb: 2W3L): (a) Bcl-2/Bcl-xL showing the binding site of DRO; (b) 3D binding mode of DRO into Bcl-2, a receptor shown as the H-bond surface; (c) 3D binding mode of the redocked DRO overlaid with the cocrystallized ligand (yellow sticks) into the active site of Bcl2; (d) 2D binding mode of the cocrystallized DRO showing different types of interactions with amino acids in Bcl2.

In the current study, compounds **3a-g** induced apoptosis and repressed Bcl-2 gene expression in HepG2 and/or MCF-7. To investigate the ability of these compounds to bind to and inhibit Bcl-2, a comparative molecular docking study was performed. The aim of this study was to assess the potential binding affinities, modes, and interactions of the new compounds against those of a selective Bcl-2 inhibitor [35]. The crystal structure of Bcl-2/Bcl-xL bound to the DRO inhibitor (pdb: 2W3L) [26] was used in this study (Figure 11(a)). The binding mode of DRO into Bcl-2 was also illustrated (Figure 11(b)). Investigation of the inhibitory activity of DRO against Bcl-2 revealed IC_50_ values of 0.03 and 0.10 *μ*M against Bcl-2 16me and Bcl-2 26me, respectively [35]. Accordingly, DRO was used as a reference drug in this study.

Validation of the docking study was initially performed, where DRO was redocked into Bcl-2 using AutoDock 4.2 [34]. The results of this validation revealed a binding free energy (Δ*G*_*b*_) of -9.67 kcal/mol for the best fitting conformation of DRO. Investigation of the binding mode of DRO revealed superposition with the cocrystallized ligand with RMSD of 0.79 Å (Figure 11(c)). Analysis of the binding interactions of DRO revealed one carbon-hydrogen bond and one electrostatic (pi-anion) interaction with Asp70. In addition, multiple hydrophobic (pi-sigma/alkyl, pi-pi T-shaped, and alkyl) interactions were also observed between DRO and amino acids into Bcl-2.

Compounds **3a-g** were also docked into the active site of Bcl-2. The results revealed binding free energies in the range of -5.73 to -7.57 kcal/mol, where **3g** exhibited the highest affinity toward Bcl-2. In addition to the multiple hydrophobic interactions, the new compounds **3a-g** also showed 1-5 hydrogen bonds of the conventional/carbon types with amino acids in Bcl-2 (Table 4).

**Figure 12:**
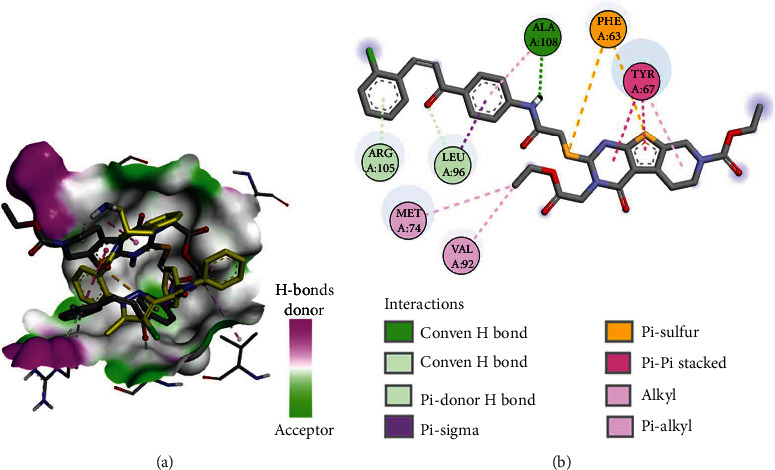
Binding mode/interactions of **3a** (shown as sticks colored by element) in Bcl-2: (a) 3D binding mode of **3a** overlaid with the cocrystallized DRO (yellow sticks) into the active site of Bcl-2; (b) 2D binding mode of **3a** showing the hydrogen bonds and electrostatic and hydrophobic interactions with amino acids in Bcl-2.

Among the new compounds, **3a** displayed a binding free energy of (Δ*G*_*b*_) of -5.94 kcal/mol toward Bcl-2 compared to -9.67 kcal/mol for DRO. Analysis of the best fitting conformation of **3a** revealed partial superposition of the thieno[2,3-*d*]pyrimidine moiety with the tetrahydroisoquinoline moiety in DRO. Investigation of the binding interaction of **3a** revealed one conventional hydrogen bond with Ala108, one carbon hydrogen bonds with Leu96, and one pi-donor bond with Arg105. Like DRO, compound **3a** exhibited similar hydrophobic interaction with Tyr67, Met74, and Val92 (Figure 12).

**Figure 13:**
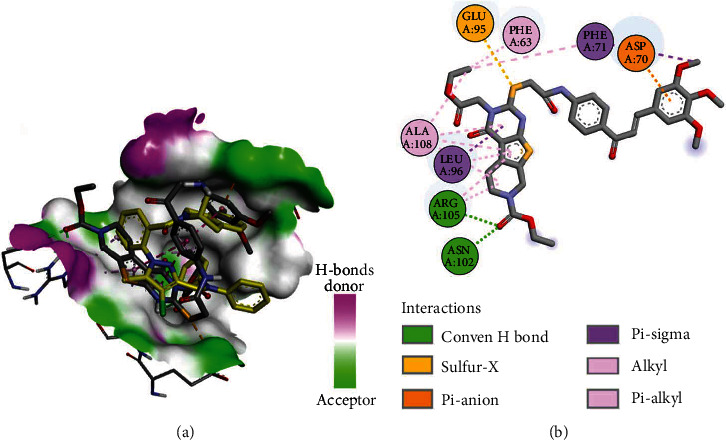
Binding mode/interactions of **3b** (shown as sticks colored by element) in Bcl-2: (a) 3D binding mode of **3b** overlaid with the cocrystallized DRO (yellow sticks) into the active site of Bcl-2; (b) 2D binding mode of **3b** showing the hydrogen bonds and electrostatic and hydrophobic interactions with amino acids in Bcl-2.

Compound **3b** also displayed a binding free energy of -5.73 kcal/mol toward Bcl-2. Analysis of the best fitting conformation of **3b** revealed superposition of the pyrimidine ring with the pyrazole ring in DRO, where the ethyl ester moiety in **3b** occupied the position of one of the two phenyl rings of the diphenylamine moiety in DRO. The trimethoxyphenyl moiety in **3b** is also superposed with the tetrahydroisoquinoline in DRO (Figure 13).

**Figure 14:**
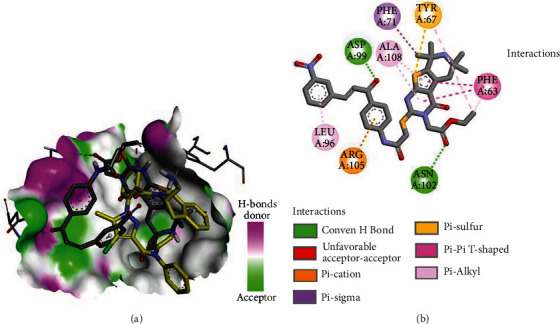
Binding mode/interactions of **3g** (shown as sticks colored by element) in Bcl-2: (a) 3D binding mode of **3g** overlaid with the cocrystallized Pro (yellow sticks) into the active site of Bcl-2; (b) 2D binding mode of **3g** showing different types of interactions with amino acids in Bcl-2.

Like DRO, compound **3b** exhibited one electrostatic interaction with Asp70. The two compounds also exhibited similar hydrophobic interactions with the same amino acids (Phe63, Phe71, Leu96, and Ala108). In addition, **3b** showed two additional conventional hydrogen Asn102 and Arg105 (Figure 13). Moreover, compound **3g** showed different types of interactions with amino acids in Bcl-2 (Figure 14).

The 2/3D binding modes and interactions of the remaining compounds (**3c-f**) are provided in the supplementary data (Figures S1–S4).

In conclusion, the results of the docking analysis revealed a relatively moderate binding affinity of the seven compounds toward the antiapoptotic protein, Bcl-2. Among these derivatives, **3g** showed the highest binding free energy. The new compounds also exhibit different types of binding interaction including hydrogen bonds and electrostatic and hydrophobic interactions.

### 3.15. ADME Study

The physicochemical properties related to drug-likeness of compounds **3a-g** were calculated using SwissADME (http://www.swissadme.ch/) [45] and Molsoft L.L.C (http://molsoft.com/mprop/). This study was aimed at evaluating the physicochemical properties of the new compounds against those of the selective Bcl-2 inhibitor DRO and the FDA-approved Bcl-2 inhibitor venetoclax. The results are presented in Table 5. The detailed results of the ADME study are provided in Supplementary 4 (Tables S1–S18).

The results of the ADME study revealed that hits **3a-g** have molecular weights (MWs) in the range of 645.68-750.84 Daltons, compared to DRO (MW = 576.09 Da) or venetoclax (MW = 868.44 Da). In addition, many of the small-molecule Bcl-2 inhibitors showed also molecular weights > 500 [46].

The designed compounds also showed lower MVs in the range of 650.41-764.06 Å^3^, which lay in between those of DRO (559.71 Å^3^) and venetoclax (854.36 Å^3^).

Compounds **3a-g** displayed calculated logP (mlogP) values in the range of 1.15-2.95, which was either equal to or lower than the mlogP value of venetoclax (2.95). These results also indicate that all the new compounds (**3a-g**) have lower lipophilicity than DRO (4.76).

The new compounds have a total number of 13-18 rotatable bonds compared to 14 for venetoclax. The new compounds also have 8-11 hydrogen bond acceptor (H_A_) compared to 9 for venetoclax, while the hydrogen bond donors (H_D_) in the new compounds were less than venetoclax.

Considering the rule of five of Lipinski which stated that the orally active drug should have no more than one violation from this rule [47]. The results in Table 5 showed that all the new compounds have two violations like DRO and venetoclax.

The physicochemical properties related to bioavailability and drug-likeness scores (DLSs) were also calculated for compounds **3a-g**. The results are presented in Table 6.

The new compounds **3a-g** showed that log *S* values are in the range of -5.64 to -6.99 compared to DRO (-7.14) and venetoclax (-9.78). They also showed TPSAs in the range of 175.76-236.26 Å^2^ compared to 183.09 for venetoclax (Table 6).

All the new compounds exhibited a similar bioavailability score of 0.17, which was equal to that of venetoclax. The fraction of the new compounds that could be absorbed from the GIT was calculated according to the previous method [47]. The calculated fractions of the new compounds **3a-g** that could be absorbed from GIT were in the range of 27.49%-48.36% compared to 45.83% for venetoclax (Table 6).

The results of the ADME study revealed that the drug-likeness scores (DLSs) are in the range of 0.17-1.68 for compounds **3a-g** compared to DRO (0.38) and venetoclax (0.57). These results also indicate that compounds **3a-g** have DLSs higher than those of DRO and venetoclax (Table 6).

### 3.16. Oxidant/Antioxidant Status in HepG2 Cells and MCF-7 Cells

**Figure 15:**
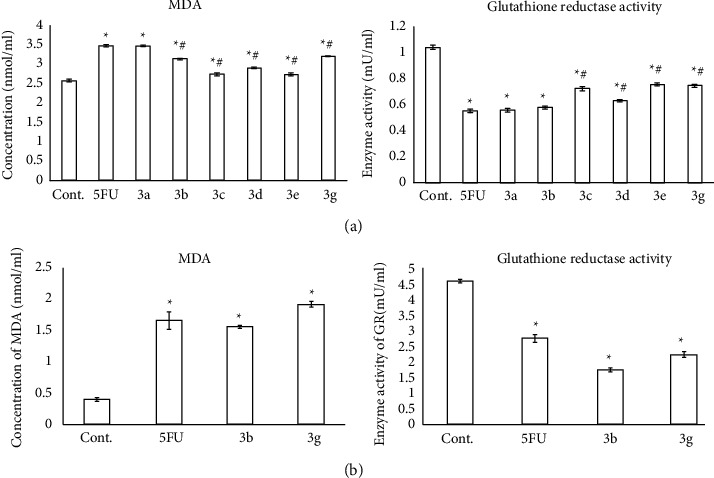
Determination of MDA and GR activity after treatment of the HepG2 cell line (a) with IC50 of some newly synthesized chalcone-thienopyrimidines (3**a**, 3**b**, 3**c**, 3**d**, 3**e**, and 3**g**) and the MCF-7 cell line (b) with IC50 of some newly synthesized chalcone-thienopyrimidine (3**a** and 3**g**) derivatives in comparison to the traditional anticancer drug 5-FU. Data were expressed for each bar as mean ± standard error of mean. ^∗^*P* ≤ 0.05 from control, ^#^*P* ≤ 0.05 from 5FU.

All tested compounds induced a significant increase (*P* < 0.05) in the MDA levels together with a marked reduction of GR activity in the HepG2 cell line compared to control cells (Figure 15(a)).

Similarly, significant elevation of MDA along with a significant decline of GR activity was noticed in MCF-7 cells exposed to **3b** and **3g** compounds in comparison to untreated cells (*P* < 0.05) (Figure 15(b)).

## 4. Discussion

As a step to progress in the field of medicine, several trials were made to join many biological activities with different moieties to develop novel compounds that have powerful anticancer effects [48]. Therefore, we synthesized novel chalcone-thienopyrimidine conjugates to develop efficient anticancer candidates. In our manuscript, we used 5-flurouracil as a standard anticancer chemotherapeutic reference which has been used in several studies dealing with the assay of the anticancer activities of thienopyrimidine [49] and other substituted pyrimidine derivatives [50]. Selection of 5-flurouracil is based on its mechanism of action that has been attributed to apoptosis induction in cancer cells [51].

The disturbance in the balance between cell death and cell division is the main eliciting factor of cancer. Apoptosis, autophagy, and necrosis are variant processes that develop cell death. Apoptosis is the physiological pathway through it, and programmed cell death occurs. Any disturbance in the process of apoptosis usually develops cancer. Thus, most of anticancer therapies were found to act mechanistically *via* induction of apoptosis [52].

During apoptosis, different biochemical markers were produced in a sequential manner. These markers include phosphatidylserine externalization, the release of proapoptotic proteins, and caspase activation. To elucidate the mechanism by which these compounds exert their anticancer activities *in vitro*, different assays were performed including MTT, cell cycle analysis, apoptosis, and molecular expressions of antiapoptotic protein Bcl-2 as well as the proapoptotic Bax, caspase-3, caspase-9, and oxidant/antioxidant markers.

The MTT assay was performed to assess the viability of cells when subjected *in vitro* to the novel chalcone-thienopyrimidine derivatives. The current results showed that only **3b** and **3g** compounds at their IC_50_ values (Figure 3(b)) exhibited promising antiproliferative potential against MCF-7 cells. On the other side, the IC_50_ of the six compounds (**3a**, **3b**, **3c**, **3d**, **3e**, and **3g**) provoked marked cytotoxic effects against the HepG2 cell line compared to 5-FU, which is one of the well-known anticancer agents (Figure 3(a)). The compound **3f** had the lowest cytotoxic activity against HepG2 and MCF-7 cell lines. The potent compounds were considered for further mechanistic studies of apoptosis and oxidative stress.

The discrepancies in the IC_50_ values might be ascribable to several issues such as the structure and functionality of the ring system and the genetic and biochemical background of the cell lines. The obvious potency of **3b** and **3g** compounds as promising anticancer agents against HepG2 and MCF-7 cell lines was attributed to the presence of 3-methoxy-donating function groups on chalcone moiety that exhibited remarkable anticancer activity (~2.5 times more activity) compared to compound **3g** containing an electron-withdrawing function group (NO_2_) on chalcone moiety.

Several reports addressed the *in vitro* potency of numerous synthesized chalcones against cancers of different organs including the lung, colon, and breast [53]. Bagul et al. [54] found that chalcone-linked pyrazoloij1,5-a] pyrimidine hybrids induced antiproliferative actions when incubated *in vitro* with MDA-MB-231 (breast cancer), -549 (lung cancer), and DU-145 (prostate cancer) cells and that the results were comparable with the used reference treatment (erlotinib). Moreover, thiopyrimidines were studied against leukemia and colon and breast cell lines, and the results indicated promising antitumor activities [55]. In addition, some novel morpholinylchalcone (the building blocks for the formation of a series of pyridopyrimidinethiones and pyrido[2,3-d][1,2,4]triazolo[4,3-a]pyrimidin-5(1H)-ones) synthesized compounds were observed to induce significant anticancer actions equivalent to the standard treatment cisplatin when applied *in vitro* against A-549 and HepG-2 cells [56]. Further, many synthesized 2-thiopyrimidine/chalcone derivatives have been noticed to have remarkable antitumor effects against various cancer cell lines including MCF-7 [11].

Cell cycle analysis of the tested novel compounds was evaluated to elucidate the molecular mechanism of antiproliferation induction in HepG2 and MCF-7 cell lines. Analysis of the cell cycle that was accomplished by propidium iodide (PI) staining in Figure 4 showed arresting of the cell cycle of the HepG2 cell line at different phases by application of the newly synthesized six tested compounds **3a**-**e** and **3g**. In this regard, the arrest in G2/M was observed with compounds **3a**, **3e**, and **3g**, while the arrest in pre-G1 phases was accompanied by compounds **3a**, **3b**, **3d**, **3e**, and **3g**. Worthwhile, compound **3c** induced a significant increase in the G1 phase cell population up to 55.32%. However, compound **3d** induced a significant increase in the S phase cell population. On the other side, in the MCF-7 cell line, compounds **3b** and **3g** significantly induced G1 and pre-G1 arrest (Figure 5). A similar data was achieved when some benzimidazole-chalcone derivatives were applied in MCF-7 cell lines [57] and thyroid carcinoma (BHT-101) cells treated with 50 *μ*M curcumin [58]. The antimitotic action of chalcones *via* arresting the cell proliferation at G2/M was first documented almost 20 years ago [59].

The results of quantitative estimation of apoptosis given by Annexin V-FITC/PI staining with flow cytometry (Figures 6 and 7) revealed that the HepG2 cell line treated with all tested compounds except **3c** showed a significant induction of total apoptosis when compared to control (*P* < 0.05). The compounds **3a**, **3b**, and **3g** significantly exhibited early and late apoptosis. Moreover, none of the tested compounds displayed a necrotic effect compared to the control (*P* > 0.05) (Figure 6). The treatment of MCF-7 cells with compounds **3a** and **3b** showed a significant increase in the total, early, and late apoptosis as well as necrosis in comparison to untreated cells (*P* < 0.05) together with enhancing the phosphatidylserine (Annexin V-positive cells) translocation. In line with these observations, some chalcone analogs showed a significantly higher percentage of apoptotic cells in HepG2 [60] and MCF-7 cells [61] than in the control cells. Takac et al. [62] reported that chalcones halted the proliferation of cancer cells *via* arresting the cell cycle. These anticancer activities of chalcone analogs might be attributed to the identity in the structure of these compounds with 5-FU as the thiopyrimidine ring in these compounds is an analogue to the pyrimidine ring in 5-FU. In this concern, 5-FU was found to inhibit thymidylate synthase, preventing DNA synthesis and inducing apoptosis [63]. Thus, the apoptotic changes that were observed in the cells of the present study might be attributed to the inhibition of DNA synthesis and the subsequent cell cycle arrest.

Apoptosis refers to the physiological mechanism that involves many signaling pathways, provoked by different stimuli primarily oxidative stress [64]. Apoptosis is induced intrinsically when the subclasses of the Bcl-2 protein family interact with the outer mitochondrial membrane. BH3 (Bcl-2 homology 3) proteins are the provoking key of apoptotic signals [65]. BH3 proteins are activated by cytotoxic stress which causes alterations in the permeability of mitochondrial membrane *via* stimulation of Bax and BAK that results in the liberation of cytochrome c (apoptogenic compound) [66]. Cytochrome c is the main activator of caspase-9 which in turn leads to the activation of both caspase-3 and caspase-7 [67], causing a cascade of proteolytic activities that lead to apoptosis [68].

The aforementioned apoptotic mechanism is inhibited by Bcl-2 proteins that interact with BH3-only proteins and accordingly inhibit Bax and BAK [65]. However, cancer cells resist apoptosis by downregulation of Bax and upregulation of Bcl-2. To observe the molecular mechanism *via* which chalcone-thienopyrimidine derivatives exert their antiproliferative activity, we investigated the effect of the most active synthesized chalcone-thienopyrimidine derivatives on the abundance of the specific genes responsible for the cell cycle and apoptosis (Bcl-2, Bax, caspase-3, and caspase-9) in both HepG2 and MCF-7 cells.

Many chalcones have been observed to target the mitochondrial pathway as a means to induce apoptosis [69]. In the HepG2 cell line, all tested compounds except **3c** have been shown to produce significant altitude in the Bax/Bcl-2 ratio (*P* < 0.05) in comparison to control. Caspase-3 levels were remarkably elevated with all tested compounds, while those of caspase-9 were raised with all tested compounds except **3e**. In the MCF-7 cell line, compounds **3b** and **3g** were associated with significant increments in proapoptotic genes (Bax, caspase-3, and caspase-9) and remarkable decrements in the antiapoptotic Bcl-2 in comparison to control (*P* < 0.05). In addition, there was a positive correlation between cytotoxic efficacy of the compounds and apoptosis in both HepG2 and MCF-7 cell lines.

These results support the notion that the mechanism of antitumor activity by these novel chalcone-thienopyrimidine derivatives includes the intrinsic (mitochondrial) apoptotic pathway. In accordance with our results, chalcones have been demonstrated to activate Bak and Bax and inhibit Bcl-2 [70], as well as activate caspase-9. Deeb et al. [71] clarified that a chalcone (xanthohumol) enhanced both intrinsic and extrinsic apoptotic pathways. Similarly, other studies demonstrated that chalcones induced apoptosis in the MCF-7 cell line by both pathways [72]. Moreover, recent studies had shown that chalcones also function as an apoptotic regulator in human lung and hepatic cancer cells and restrain cancer cell metastasis [73]. Further, the indole-chalcone-based benzopyran chalcones when used *in vitro* against cancer cells resulted in antiproliferation of the cells by eliciting DNA nick-sealing activity *via* inhibition of DNA ligases [74]. In addition, the proliferation of K562 was inhibited by chalcone derivatives through induction of apoptosis [75]. The chemical structures of chalcones seem to play a pivotal role in detecting their molecular targets. The apoptotic effect of the tested compounds was elucidated by upregulation of Bax, caspase-3, and caspase-9 and downregulation of Bcl-2. The aforementioned results were supported by the findings of the molecular docking analysis into Bcl-2 that exhibited moderate binding affinity of the seven tested compounds for Bcl-2 comparable to one of the well-known Bcl-2 inhibitors, DRO [35, 44]. Among them, **3g** showed the highest binding free energy. The remaining tested compounds showed different types of binding interactions including hydrogen bonds and electrostatic and hydrophobic interactions.

Generation of free radicals during oxidation results in a chain of reactions that results in cell damage and develops an oxidative stress state that causes several chronic diseases such as cancer [76]. During oxidative stress, the redox system is altered together with the disturbance in cell proliferation and apoptosis [77]. However, chalcones possess antioxidant properties, but under certain circumstances, chalcones may act as oxidants [78], and this effect can be associated with their antitumor activity [79]. Cancer cells are highly sensitive to the prooxidants [80] due to higher concentrations of some ions and higher metabolic activity [81] in comparison with noncancer cells. The mechanisms through which the chalcones revealed prooxidant actions are either elevation of the superoxide levels [81], elimination of cellular glutathione [82], or formation of phenoxyl radicals [82].

Recently, it has been established that chalcones (either natural or synthetic) exert their antiproliferative activities *via* induction of oxidative stress [83]. Therefore, we investigated the impact of newly synthesized chalcone-thienopyrimidine derivatives on MDA as an indicator of lipid peroxidation and GR activity 5as one of the intracellular antioxidant defense mechanisms. All tested compounds induced a significant increase in the MDA levels together with a marked reduction of GR activity in HepG2 cells in comparison to untreated cells (*P* < 0.05). Similarly, significant elevation of MDA along with a significant decline of GR activity was noticed in MCF-7 cells exposed to **3b** and **3g** compounds in comparison to untreated cells (*P* < 0.05) (Figure 15). These findings supported the efficacy of the tested compounds for causing the oxidative stress-induced apoptosis by suppression of GR activity and increasing lipid peroxidation in cancer cells. In line with our findings, Takac et al. [62] detected significant increases in superoxide, nitric oxide, and lipid peroxide concentrations and a significant drop in GSH levels when the colorectal cancer HCT116 cell line was treated with chalcone. These results were echoed in similar studies [84, 85]. Furthermore, as venetoclax, compounds **3a-g** exhibited 2 violations from Lipinski's rule. However, the findings of the ADME study also indicated higher drug-likeness scores for compounds **3a-g** than for venetoclax.

The present study suggested that the newly synthesized chalcone-thienopyrimidine derivatives had the ability to generate oxidative stress, subsequently inducing apoptosis in MCF-7 and HepG2 cells through the intrinsic pathway as a possible mechanism of their anticancer activity.

## 5. Conclusion

Novel chalcone-pyridothienopyrimidinone derivatives were synthesized when chalcones reacted with different pyridothienopyrimidine under reflux temperature. It is interesting to observe that the IC_50_ of the two compounds **3b** and **3g** showed a higher cytotoxicity than 5-FU against HepG2 and MCF-7 cell lines. The six tested compounds (**3a**-**e** and **3g**) arrested the proliferation of HepG2 and MCF-7 cell lines at different cell cycle phases. Interestingly, our results revealed that treatment of HepG2 and MCF-7 cells with the newly synthesized tested compound elicited apoptotic effects comparable to that with 5-FU. The mechanism by which the tested compounds induced apoptosis included upregulation of the proapoptotic genes (Bax, caspase-9, and caspase-3) and downregulation of the antiapoptotic Bcl-2. The data of the molecular docking analysis of **3a-g** compounds into the binding groove of the antiapoptotic protein Bcl-2 revealed relatively moderate binding free energies compared to the selective Bcl-2 inhibitor DRO. Among the new compounds, **3g** showed the highest binding free energy toward Bcl-2. Analysis of the binding interactions of **3a-g** revealed multiple hydrophobic interactions besides the hydrogen bonds and electrostatic interactions with amino acids in the active site of Bcl-2. Compounds **3a-g**, like venetoclax, revealed two violations of Lipinski's rule. However, compounds **3a-g** have greater drug-likeness scores in the ADME study than venetoclax.

Intriguingly, all tested compounds were associated with significant increases in MDA levels together with a marked reduction in GR activity in HepG2 and MCF-7 cell lines compared to control cells. This suggested that the newly synthesized chalcone-thienopyrimidine derivatives had the ability to induce anticancer activity *via* inducing oxidative stress which in turn triggered apoptosis in MCF-7 and HepG2 cells through the intrinsic pathway. Anticancer compounds act mechanistically by interfering with the cell cycle and by triggering oxidative stress-dependent apoptosis. Therefore, regulation of the cell cycle and apoptosis are suggested to be active therapeutic strategies for the development of novel therapies in oncology. The tested compounds have the potential to be taken up for further modern clinical trials after extensive pharmacodynamic, pharmacokinetic, and toxicity profile investigations, particularly against liver and breast cancer.

## Figures and Tables

**Table 1:** Primer sequences used for RT-PCR.

Parameter	Primer sequence
Bax	Forward primer: 5′-GTTTCA TCC AGG ATC GAG CAG-3′ Reverse primer: 5′-CATCTT CTT CCA GAT GGT GA-3′
Cas3	Forward primer: 5′-CTCGGTCTGGTACAGATGTCGA-3′ Reverse primer: 5′-CATGGCTCAGAAGCACACAAAC-3′
Cas9	Forward primer: 5′-CTCCAACATCGACTGTGAGAAGTT-3′ Reverse primer: 5′-GCGCCAGCTCCAGCAA-3′
Bcl-2	Forward primer: 5′-CCTGTG GAT GAC TGA GTA CC-3′ Reverse primer: 5′-GAGACA GCC AGG AGA AAT CA-3′
*β*-Actin	Forward primer: 5′-GTGACATCCACACCCAGAGG-3′ Reverse primer: 5′-ACAGGATGTCAAAACTGCCC-3′

**Table 2:** Preliminary screening of the cytotoxic efficacy of the synthesized chalcone-thienopyrimidine derivatives **3a-g** against human breast cancer cell line (MCF-7) compared to DMSO. The cytotoxic efficacy of the compounds is expressed as the mean of survival percentage ± SEM.

Compound concentration (*μ*g/ml)	**3a**	**3b**	**3c**	**3d**	**3e**	**3f**	**3g**	*DMSO*
10000	6.0 ± 0.063	17.3 ± 0.001	5.9 ± 0.002	5.8 ± 0.001	15.9 ± 0.002	4.6 ± 0.002	15.3 ± 0.000	4.2 ± 0.071
5000	6.0 ± 0.001	19.7 ± 0.002	6.4 ± 0.007	6.12 ± 0.003	16.0 ± 0.000	5.1 ± 0.003	18.9 ± 0.001	4.2 ± 0.121
2500	6.28 ± 0.003	20.1 ± 0.001	6.4 ± 0.003	6.2 ± 0.005	17.9 ± 0.002	6.2 ± 0.004	19.0 ± 0.001	6.8 ± 0.257
1250	9.8 ± 0.003	20.3 ± 0.001	7.4 ± 0.003	8.5 ± 0.006	27.8 ± 0.021	47.5 ± 0.181	19.9 ± 0.002	11.5 ± 0.112
625	64.9 ± 0.008	20.3 ± 0.002	11.3 ± 0.026	26.5 ± 0.120	38.0 ± 0.030	67.1 ± 0.086	19.9 ± 0.001	15.1 ± 0.313
312.5	101.6 ± 0.022	21.0 ± 0.001	71.1 ± 0.156	35.3 ± 0.044	61.5 ± 0.112	92.0 ± 0.141	19.4 ± 0.004	26.8 ± 0.245
156.25	101.8 ± 0.284	21.3 ± 0.013	110.9 ± 0.063	103.1 ± 0.202	104.2 ± 0.026	119.7 ± 0.182	20.1 ± 0.003	102.6 ± 0.632
78.125	108.4 ± 0.084	27.7 ± 0.026	121.5 ± 0.183	113.1 ± 0.171	109.2 ± 0.084	120.2 ± 0.150	28.0 ± 0.015	113.0 ± 0.334
39.0625	129.1 ± 0.038	31.8 ± 0.009	132.0 ± 0.136	132.7 ± 0.231	129.1 ± 0.033	122.6 ± 0.121	81.0 ± 0.009	116.8 ± 0.277
19.53125	124.4 ± 0.079	83.2 ± 0.022	140.3 ± 0.197	133.2 ± 0.116	123.4 ± 0.070	127.7 ± 0.180	96.4 ± 0.003	129.7 ± 0.299
9.765625	132.9 ± 0.132	102.6 ± 0.015	135.4 ± 0.066	146.0 ± 0.065	131.6 ± 0.132	132.2 ± 0.214	98.2 ± 0.023	130.2 ± 0.226
4.8828125	150.0 ± 0.171	104.9 ± 0.016	149.3 ± 0.082	133.8 ± 0.227	147.1 ± 0.051	149.7 ± 0.082	105.3 ± 0.032	145.8 ± 0.072

**Table 3:** Preliminary screening of the cytotoxic efficacy of the synthesized chalcone-thienopyrimidine derivatives 3a-g against human liver cancer cell line (HepG2) compared to DMSO. The cytotoxic efficacy of the compounds is expressed as the mean of survival percentage ± SEM.

Compound concentration (*μ*g/ml)	**3a**	**3b**	**3c**	**3d**	**3e**	**3f**	**3g**	*DMSO*
10000	18.5 ± 0.001	12.1 ± 0.002	18.0 ± 0.001	18.4 ± 0.002	14.1 ± 0.002	10.7 ± 0.004	12.3 ± 0.002	10.6 ± 0.018
5000	19.1 ± 0.001	12.1 ± 0.001	18.8 ± 0.001	18.5 ± 0.003	16.8 ± 0.003	11.6 ± 0.002	12.5 ± 0.001	11.2 ± 0.001
2500	19.5 ± 0.001	12.2 ± 0.001	19.0 ± 0.002	19.4 ± 0.004	17.4 ± 0.002	11.7 ± 0.009	12.7 ± 0.002	11.6 ± 0.028
1250	20.5 ± 0.004	12.7 ± 0.002	20.1 ± 0.004	19.5 ± 0.001	18.4 ± 0.003	12.6 ± 0.019	12.5 ± 0.001	14.4 ± 0.028
625	21.7 ± 0.012	12.9 ± 0.006	20.6 ± 0.003	20.0 ± 0.001	18.9 ± 0.001	15.3 ± 0.005	13.0 ± 0.003	14.4 ± 0.061
312.5	28.4 ± 0.009	13.3 ± 0.004	21.1 ± 0.004	20.5 ± 0.002	19.8 ± 0.005	77.8 ± 0.071	14.1 ± 0.002	44.4 ± 0.012
156.25	40.1 ± 0.021	16.2 ± 0.004	22.1 ± 0.011	20.5 ± 0.002	21.1± 0.012	112.0 ± 0.460	14.5 ± 0.003	103.6 ± 0.051
78.125	66.9 ± 0.018	17.3 ± 0.007	62.4 ± 0.051	31.0 ± 0.017	21.4 ± 0.073	115.9 ± 0.157	17.5 ± 0.002	109.4 ± 0.040
39.0625	73.8 ± 0.019	18.4 ± 0.006	63.0 ± 0.030	91.2 ± 0.138	45.1 ± 0.072	119.1 ± 0.122	64.1 ± 0.038	113.6 ± 0.052
19.53125	74.2 ± 0.012	23.2 ± 0.012	69.8 ± 0.027	99.2 ± 0.026	91.9 ± 0.060	144.7 ± 0.140	97.8 ± 0.053	118 ± 0.045
9.765625	87.8 ± 0.087	67.9 ± 0.048	81.6 ± 0.027	111.0 ± 0.041	114.3 ± 0.141	149.1 ± 0.106	115.9 ± 0.098	174.4 ± 0.129
4.8828125	103.5 ± 0.033	110.1 ± 0.046	91.5 ± 0.013	123.0 ± 0.026	127.5 ± 0.033	149.7 ± 0.200	131.3 ± 0.033	183.0 ± 0.078

**Table 4:** Docking results of **3a-g** into Bcl-2 in comparison to its cocrystallized ligand (DRO).

Ligand	Δ*G*_*b*_^a^	*K*_*i*_^b^	HBs^c^	Amino acids involved in the hydrogen bonds
**3a**	-5.94	44.57 *μ*M	3	Leu96^∗^, Arg105^∗^, Ala108
**3b**	-5.73	62.76 *μ*M	2	Asn102 and Arg105
**3c**	-6.27	25.48 *μ*M	2	Asn102, Arg105
**3d**	-6.47	18.18 *μ*M	4	Asp70^∗^, Glu73^∗^, Arg88, Glu95
**3e**	-7.39	3.86 *μ*M	5	Met74^∗^, Gly77^∗^, Leu87^∗^, Arg88^∗^, Val92^∗^
**3f**	-7.41	3.71 *μ*M	1	Arg105
**3g**	-7.57	2.85 *μ*M	2	Asp99, Asn102
DRO	-9.67	81.01 nM	1	Asp70^∗^

^a^Binding free energy (kcal/mol). ^b^Inhibition constant. ^c^Number of hydrogen bonds. ^∗^Amino acids indicated by the asterisk are involved in carbon hydrogen bonds with the ligands.

**Table 5:** Physicochemical properties and DLSs of compounds **3a-g**, DRO, and venetoclax.

Comp.	Physicochemical properties						Lipinski's rule	
	MW	MV	mlogP	NorO	H_A_	H_D_	Obey	Comments
**3a**	695.20	683.62	2.95	15	8	1	No	2 violations: MW > 500, NorO > 10
**3b**	750.84	764.06	1.59	18	11	1	No	2 violations: MW > 500, NorO > 10
**3c**	705.76	694.58	1.74	16	10	1	No	2 violations: MW > 500, NorO > 10
**3d**	690.76	720.46	1.15	16	10	1	No	2 violations: MW > 500, NorO > 10
**3e**	664.75	672.18	1.61	14	9	2	No	2 violations: MW > 500, NorO > 10
**3f**	645.68	650.41	1.55	14	9	1	No	2 violations: MW > 500, NorO > 10
**3g**	689.80	692.96	1.85	13	9	2	No	2 violations: MW > 500, NorO > 10
DRO	576.09	559.71	4.76	8	4	1	No	2 violations: MW > 500, mlogP > 4.15
LBM	868.44	854.36	2.95	14	9	3	No	2 violations: MW > 500, NorO > 10

MW: molecular weight (Da); MV: molecular volume (Å^3^); mlogP: logP calculated using the topological method implemented from Moriguchi et al. [86]; NorO: number of rotatable bonds; LBM: venetoclax.

**Table 6:** Physicochemical properties and DLSs of compounds **3a-g**, DRO, and venetoclax.

Comp.	Log *S*	Solubility	TPSA	%Abs	BS	DLS
**3a**	-6.99	7.05*e* − 05 mg/ml	190.44	43.30	0.17	1.01
**3b**	-6.66	1.66*e* − 04 mg/ml	218.13	33.75	0.17	1.35
**3c**	-6.48	2.36*e* − 04 mg/ml	236.26	27.49	0.17	0.81
**3d**	-5.82	1.06*e* − 03 mg/ml	175.76	48.36	0.17	1.42
**3e**	-6.13	4.95*e* − 04 mg/ml	202.69	39.07	0.17	1.68
**3f**	-5.64	1.48*e* − 03 mg/ml	193.89	42.11	0.17	0.82
**3g**	-6.90	8.63*e* − 05 mg/ml	218.75	33.53	0.17	0.17
DRO	-7.14	4.20*e* − 05 mg/ml	84.46	79.86	0.17	0.38
LBM	-9.78	1.44*e* − 07 mg/ml	183.09	45.83	0.17	0.57

Log *S*: 10-based logarithm of the solubility; TPSA: topological polar surface area (Å^2^); %Abs: % absorbed orally, %Abs = 109^_^ (0.345 × TPSA); BS: bioavailability score; LBM: venetoclax. DLSs were calculated using Molsoft (http://molsoft.com/mprop).

## Data Availability

All generated data in this study are included in the article and the supplementary files.

## Supplementary Materials

Supplementary 1 Figures S1–S4.

Supplementary 2 Experiment.

Supplementary 3
^1^H NMR, ^13^C NMR, and DEPT.

Supplementary 4 ADME study and Tables S1–S18.
