# Supplementary material for: Synthesis, Anticancer Assessment, and Molecular Docking of Novel Chalcone-Thienopyrimidine Derivatives in HepG2 and MCF-7 Cell Lines
Source: Oxid Med Cell Longev. 2021 Dec 28;2021:4759821. doi: 10.1155/2021/4759821 (PMC8728392; doi:10.1155/2021/4759821)
Supplement: Supplementary 1 — Figures S1–S4. [file 4759821.f1.docx]

**Supplementary figures**


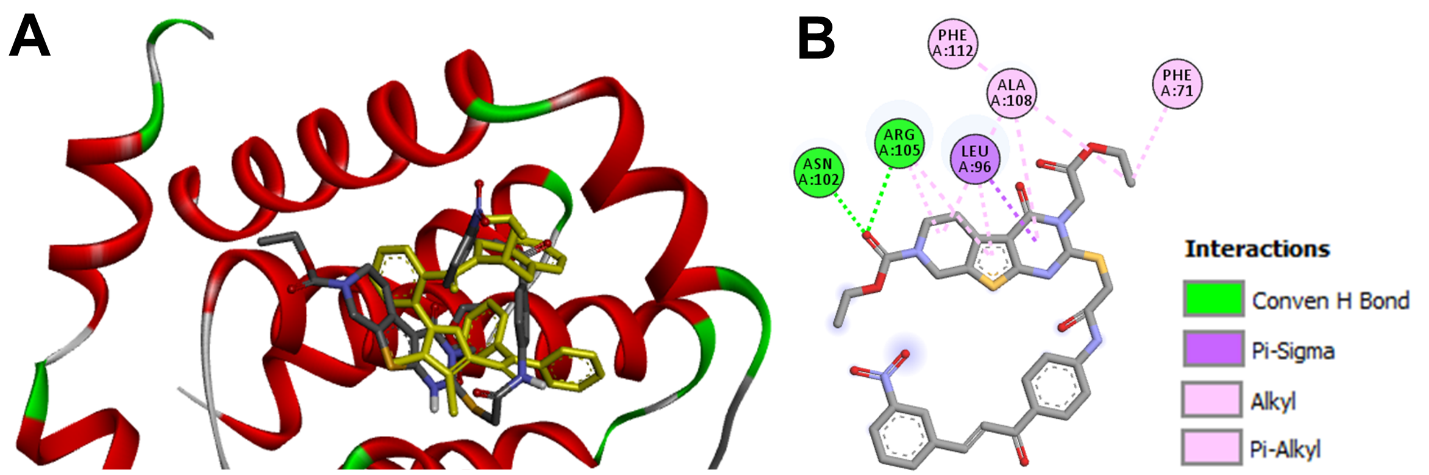


**Fig. S1.** Binding mode/interactions of **3c** (shown as sticks colored by element) in Bcl-2: A) 3D binding mode of **3c** overlaid with the co-crystallized DRO (yellow sticks) into the active site of Bcl-2; B) 2D binding mode of **3c** showing different types of interactions with amino acids in Bcl-2.


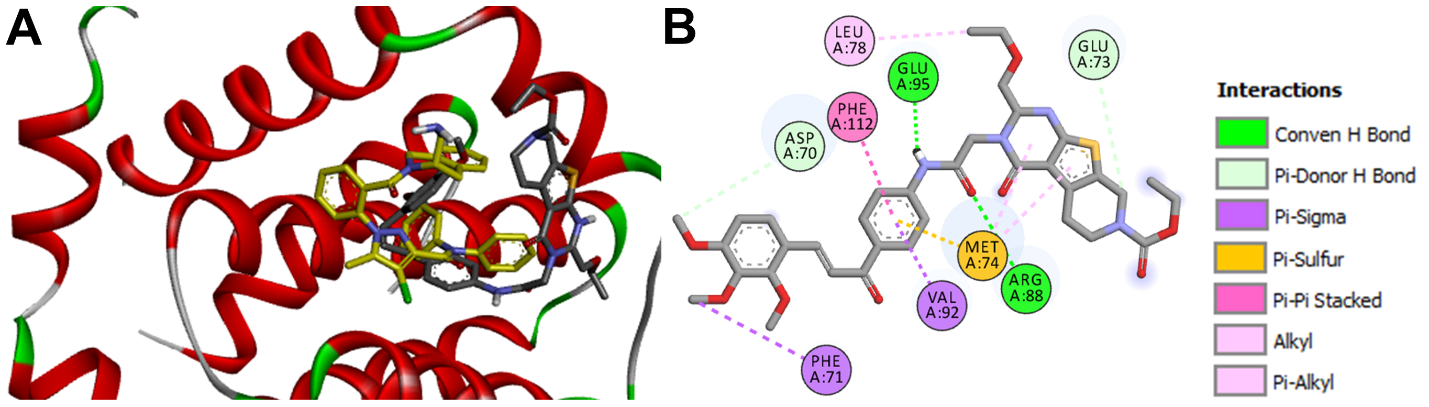


**Fig. S2.** Binding mode/interactions of **3d** (shown as sticks colored by element) in Bcl-2: A) 3D binding mode of **3d** overlaid with the co-crystallized DRO (yellow sticks) into the active site of Bcl-2; B) 2D binding mode of **3d** showing different types of interactions with amino acids in Bcl-2.


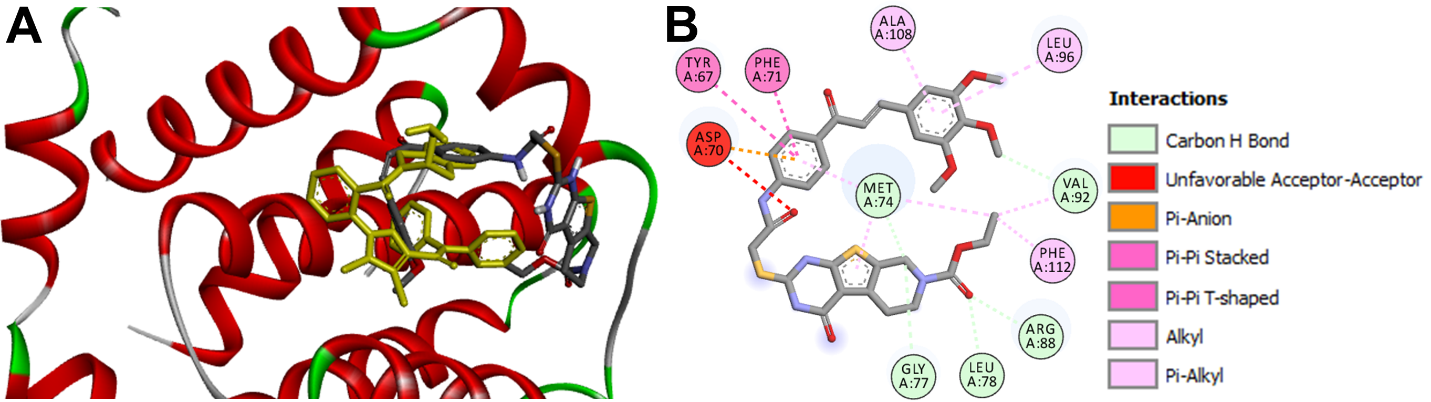


**Fig. S3.** Binding mode/interactions of **3e** (shown as sticks colored by element) in Bcl-2: A) 3D binding mode of **3e** overlaid with the co-crystallized DRO (yellow sticks) into the active site of Bcl-2; B) 2D binding mode of **3e** showing different types of interactions with amino acids in Bcl-2.


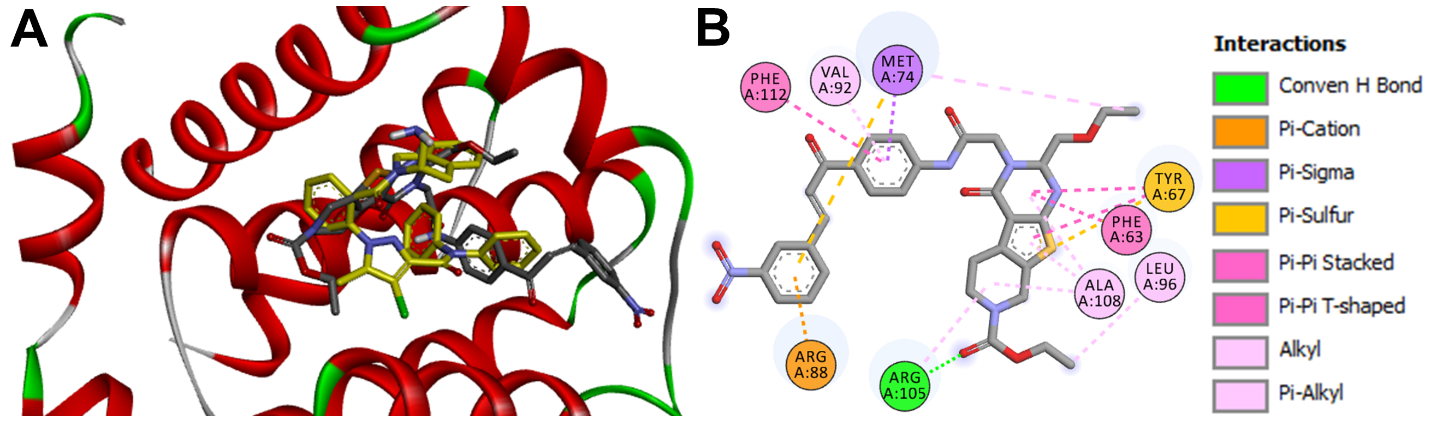


**Fig. S4.** Binding mode/interactions of **3f** (shown as sticks colored by element) in Bcl-2: A) 3D binding mode of **3f** overlaid with the co-crystallized DRO (yellow sticks) into the active site of Bcl-2; B) 2D binding mode of **3f** showing different types of interactions with amino acids in Bcl-2.
