# Supplementary material for: Synthesis, Anticancer Assessment, and Molecular Docking of Novel Chalcone-Thienopyrimidine Derivatives in HepG2 and MCF-7 Cell Lines
Source: Oxid Med Cell Longev. 2021 Dec 28;2021:4759821. doi: 10.1155/2021/4759821 (PMC8728392; doi:10.1155/2021/4759821)
Supplement: Supplementary 2 — Experiment. [file 4759821.f2.docx]

***2.5.2. MTT assay for determination of cell proliferation***

The 3-[4,5-dimethylthiazol-2-yl]-2,5-diphenyltetrazolium bromide (MTT) colorimetric assay was used to examine the sensitivity of cells to tested compounds (as anticancer drugs), as previously described by **Hussein et al. (2017).** This assay depend on the production of a purple formazan derivative from the yellow tetrazolium bromide (MTT) by mitochondrial succinate dehydrogenase in viable cells. Briefly, after harvesting the cells with 0.25% trypsin, the cell lines were suspended in DMEM. Cells were seeded in 96-well microtiter plates at a concentration of 5×10^4^ cell/well in a fresh medium and left to attach to the plates for 24 h before treatment with the tested compounds **3a-g**. Test compounds were dissolved in dimethyl sulfoxide (DMSO). After 24 hours, cells were incubated with the appropriate concentration ranges of compounds, the wells were diluted to 200 mL with fresh medium and incubation was continued for 24 hours. Three wells were used for each concentration. After 24 hours of treatment, 20 μl of MTT solution (5mg/ml) was added and subsequently the plates were further incubated for 4 hours to allow the viable cells to biotransform the yellow-colored MTT into purple formazan crystals. The optical density (O.D.) of each well was measured spectrophotometrically at 750 nm with an enzyme-linked immunosorbent assay (ELISA) microplate reader (BioTek, EL-800, USA). The percentage of cell survival was calculated as follows:

$$Percentage of survival = \frac{O.D. treated cells}{O.D. control cells} \times100$$

***2.5.4. Measurement of apoptosis using Annexin V-FITC/PI Apoptotic Assay***

Annexin V fluorescein isothiocyanate (FITC) and counterstaining with propidium iodide (PI) using the Annexin V-FITC/PI apoptosis detection kit (BD Biosciences, San Diego, CA) were employed to distinguish early and late apoptotic cells according to the manufacturer's directions. Annexin V conjugated with fluorescein isothiocyanate was used to quantify the loss of phosphatidylserine asymmetry in cell membranes involved in apoptosis, and propidium iodide can distinguish between early apoptosis, late apoptotic and necrotic cells **(Phang et al., 2016).**

Briefly, HepG2 and MCF-7 cell lines (3 × 10^5^ cells/well) were seeded in a 6-well plate and exposed to IC_50_ concentration of newly synthesized chalcone-thienopyrimidinne derivatives for 48 hours. Cells were gently washed twice with PBS accompanied by tryptic digestion (0.25% trypsin/EDTA) and washed yet again with PBS. All the cells including the floating and adherent ones were harvested, pooled and stained with 5 ml Annexin VFITC and 5 ml PI in 1× binding buffer for 15 min at room temperature in the dark. Analyses were achieved by FACS Calibur flow cytometer (BD Biosciences, San Jose, CA).

***2.8. Determination of lipid peroxidation (MDA)***

MDA has been [described](https://synonyms.reverso.net/synonym/en/described) as the product of lipid peroxidation that reacts with thiobarbituric acid to produce a red substance that is absorbed at 535 nm **(**Kei **et al., 1978).** HepG2 and MCF7 cells treated with synthetic thienopyrimidine were mixed with 2 ml of solution of trichloroacetic acid (TCA), thiobarbituric acid (TBA) and hydrochloric acid (HCl) and mixed thoroughly, and then heated in a boiling water bath. during 15 minutes. After cooling, the precipitate was removed by centrifugation at 3000 rpm for 10 minutes. Relative to the reagent blank, which measures the absorbance at 535 nm, the blank reagent contains all reagents except cells. The MDA concentration is expressed as nmol/ml using a calibration curve.

**2.9. Determination of Glutathione Reductase (GR)**

The activity of GR was determined by the decrease in absorbance caused by the oxidation of NADPH during the reduction of oxidized GSH (**Malik et al., 2019).** HepG2 or MCF-7cells were pre-treated with newly synthesized chalcone-thienopyrimidines derivatives for 24 hours, harvested and the pellet was re-suspended in twice the volume of protein extraction buffer (PEB). Cells were then vortexed vigorously for 2 min, repeating the cycle three times. Homogenate was then centrifuged at 10,000 rpm for 10 min. The supernatant was collected and was used to quantitate antioxidant enzyme GR using standard protocols. All determinations were made in at least five replicates and the values given are the average of these replicates.

.
