# Supplementary material for: Synthesis, Anticancer Assessment, and Molecular Docking of Novel Chalcone-Thienopyrimidine Derivatives in HepG2 and MCF-7 Cell Lines
Source: Oxid Med Cell Longev. 2021 Dec 28;2021:4759821. doi: 10.1155/2021/4759821 (PMC8728392; doi:10.1155/2021/4759821)

^1^H NMR, ^13^C NMR and DEPT for compound 3a

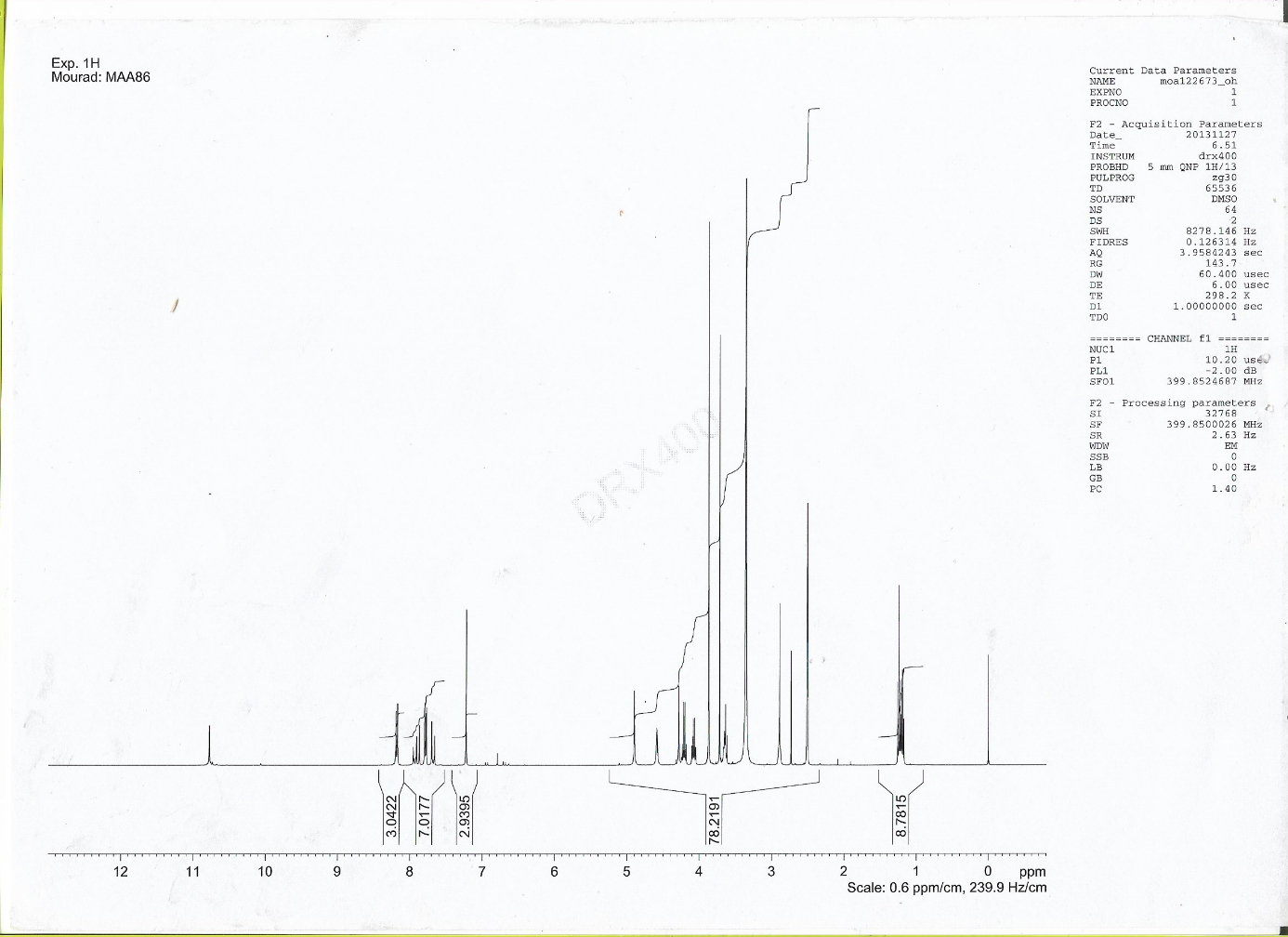


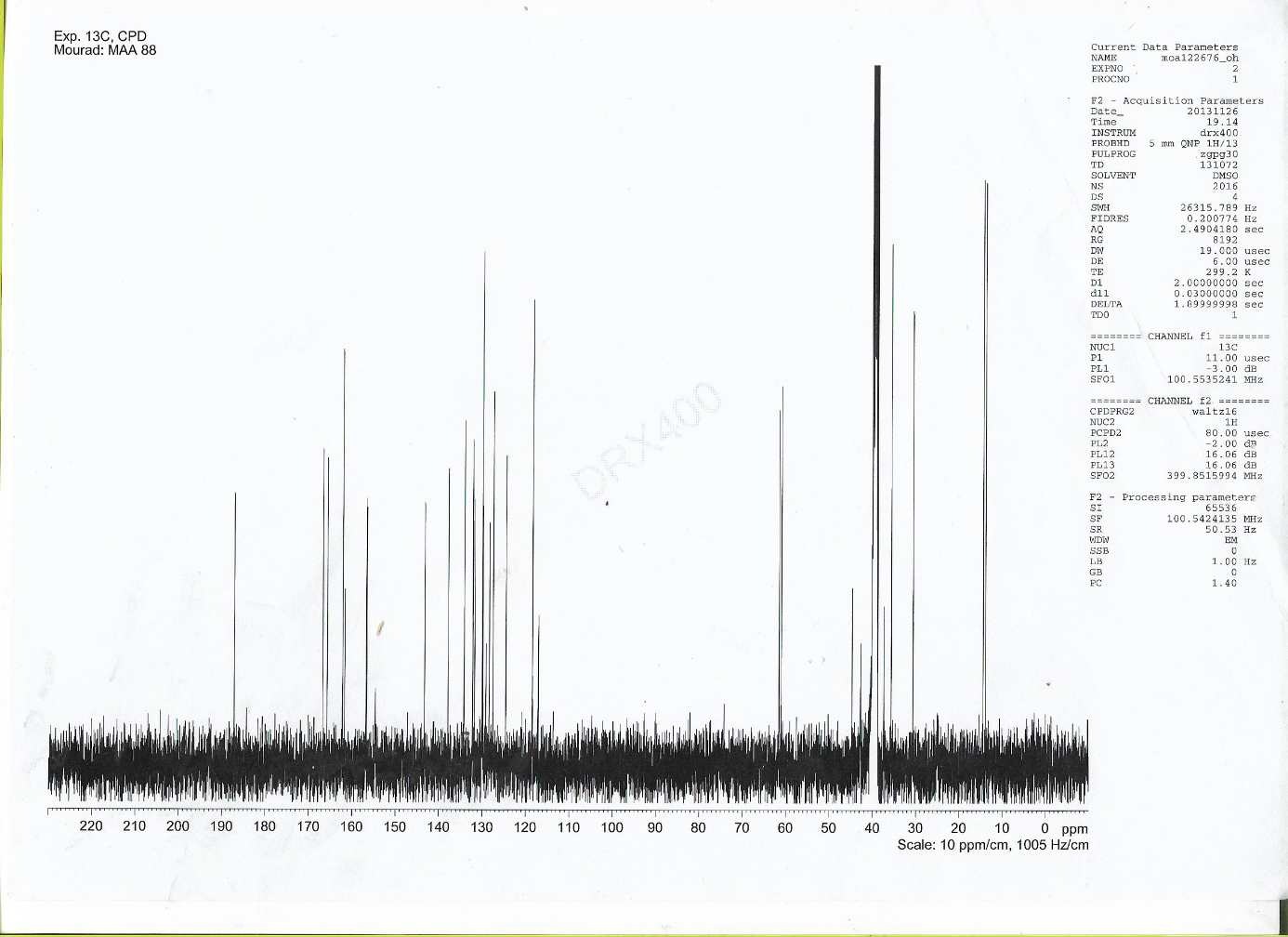


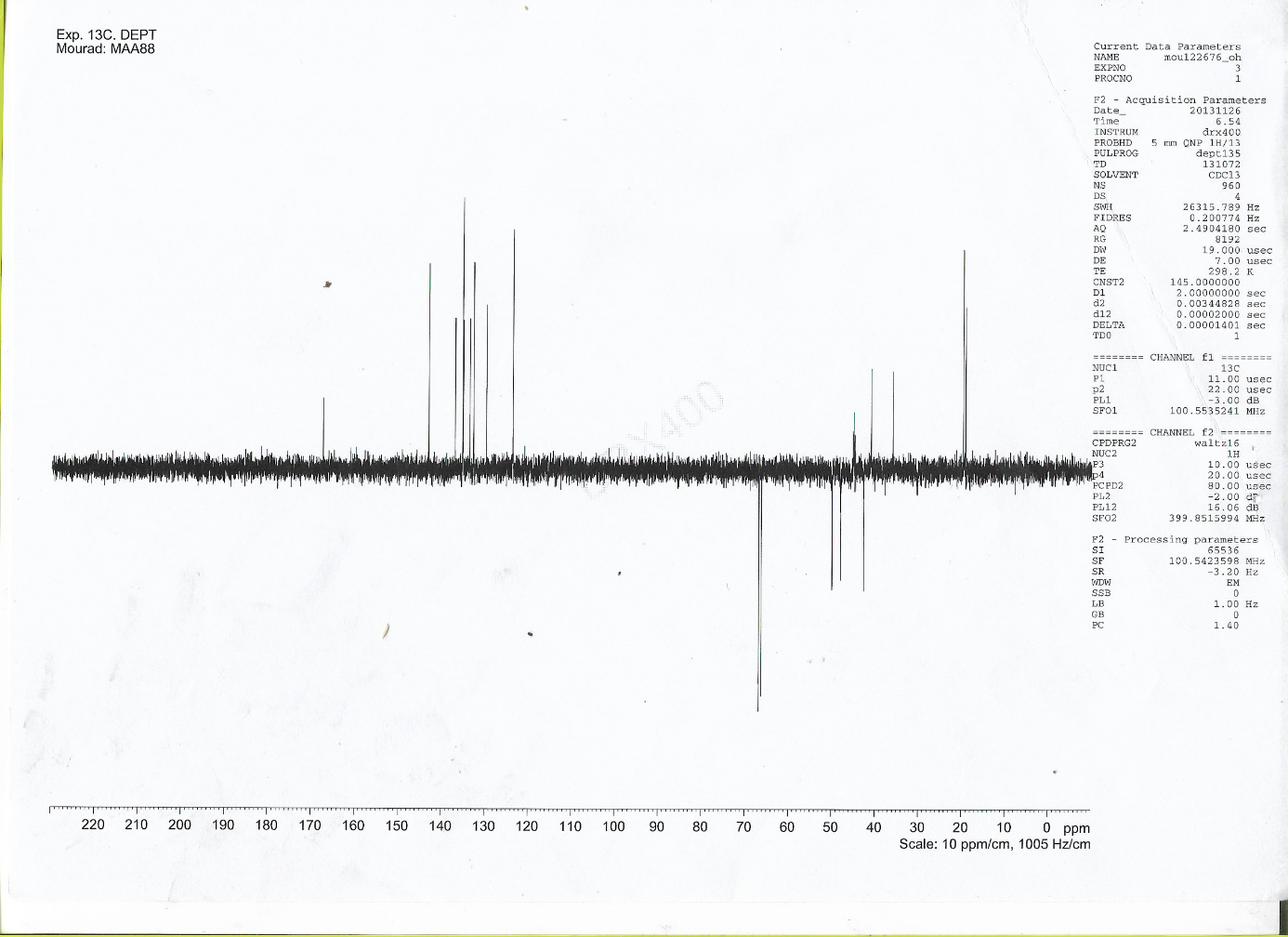


^1^H NMR, ^13^C NMR and DEPT for compound 3b

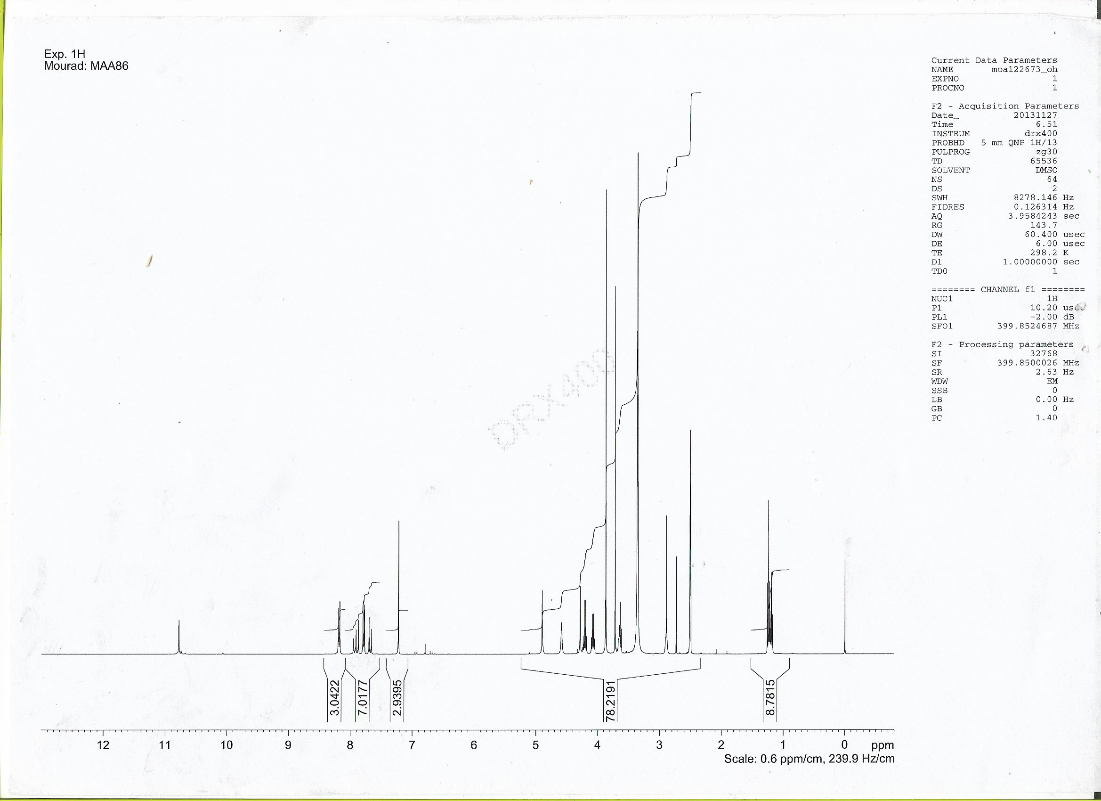


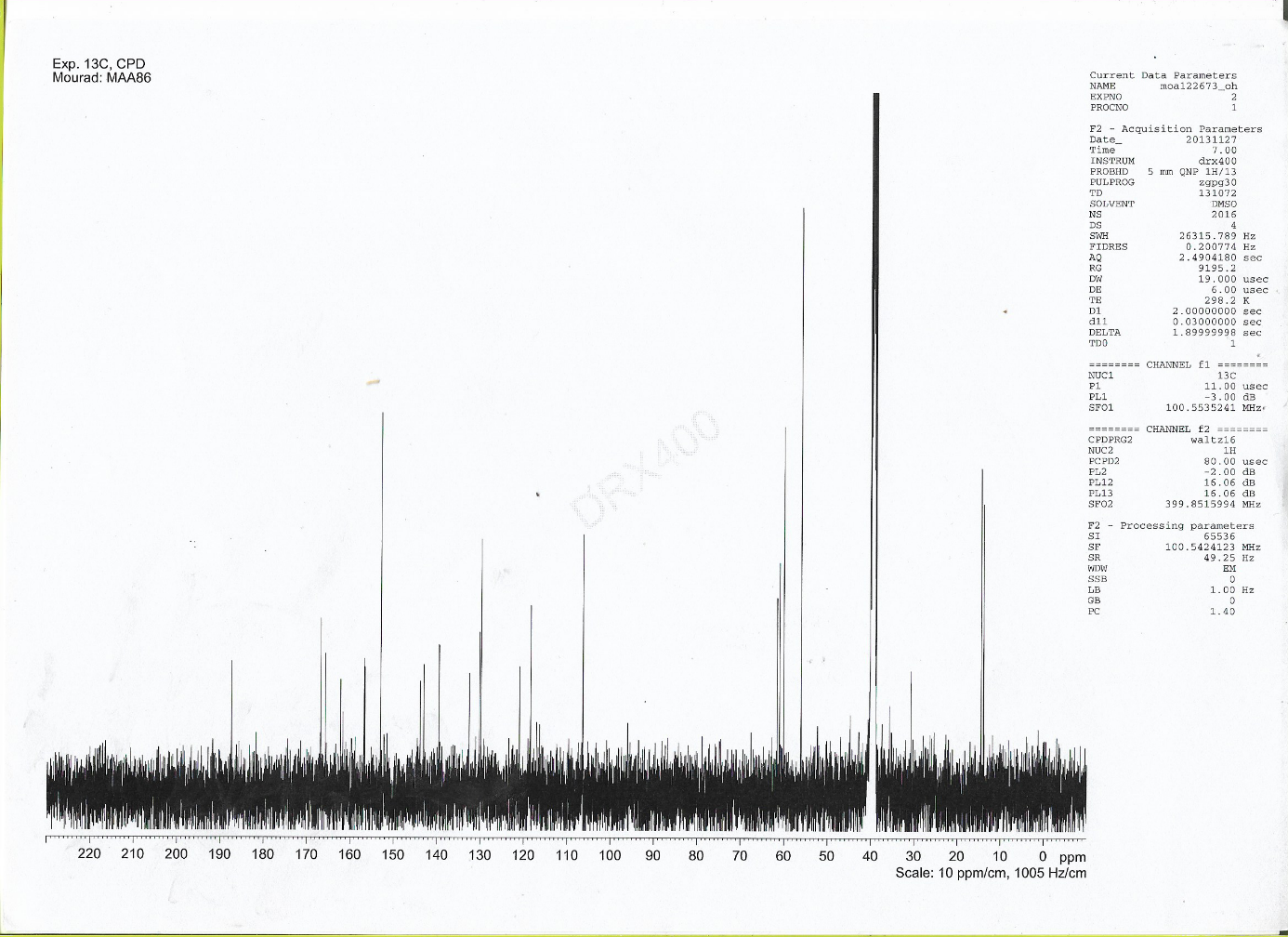


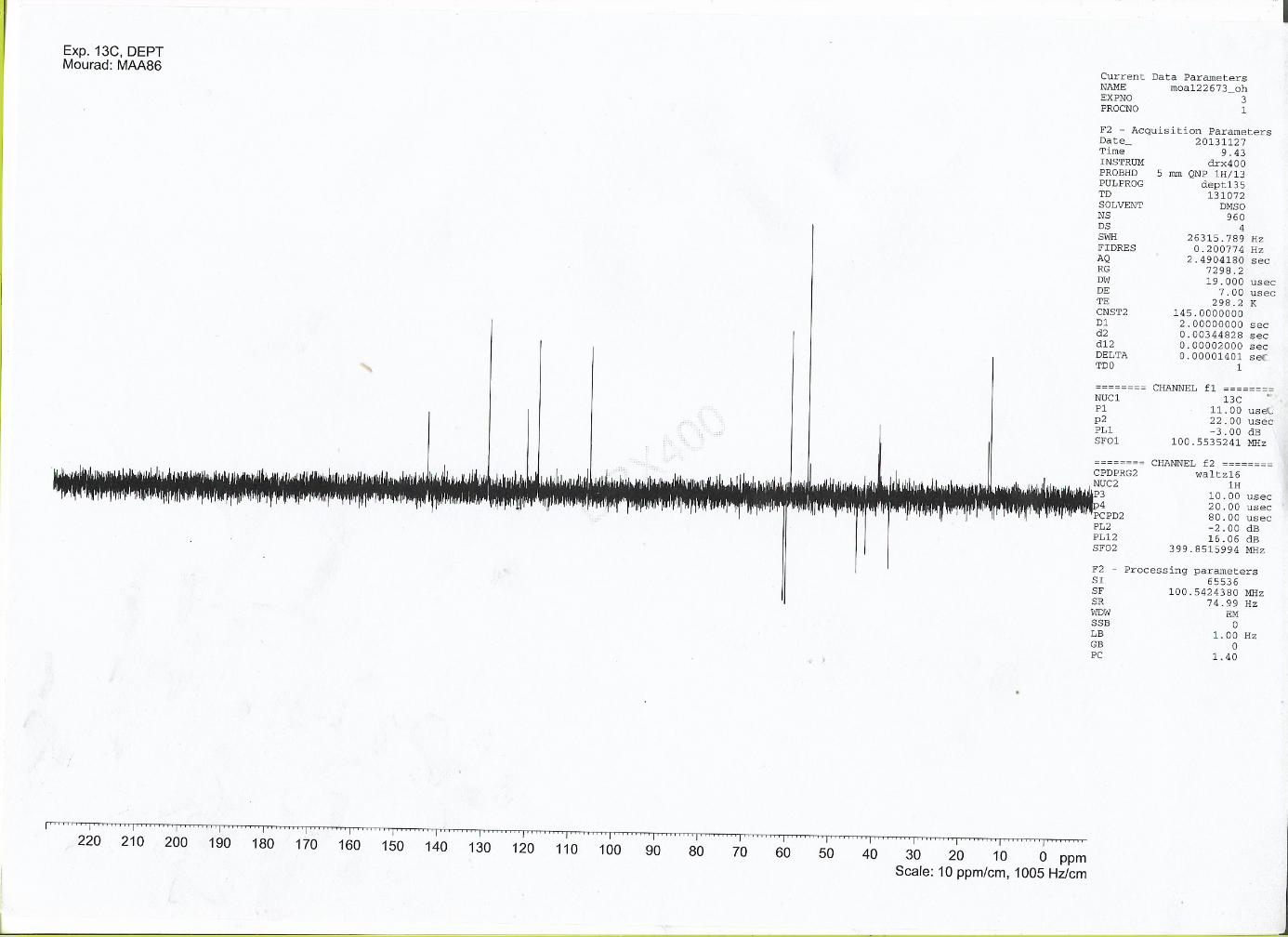


^1^H NMR, ^13^C NMR and DEPT for compound 3e

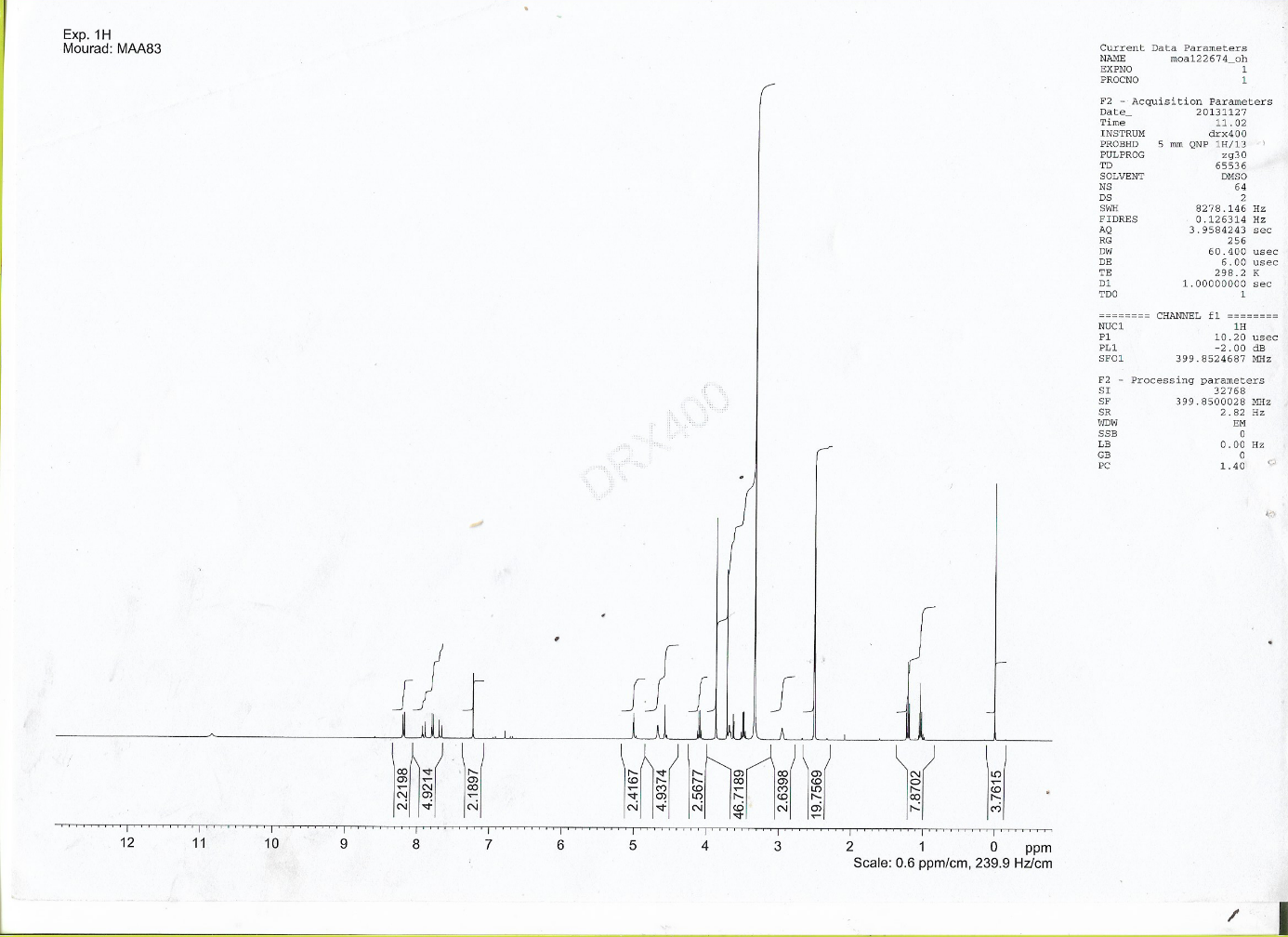


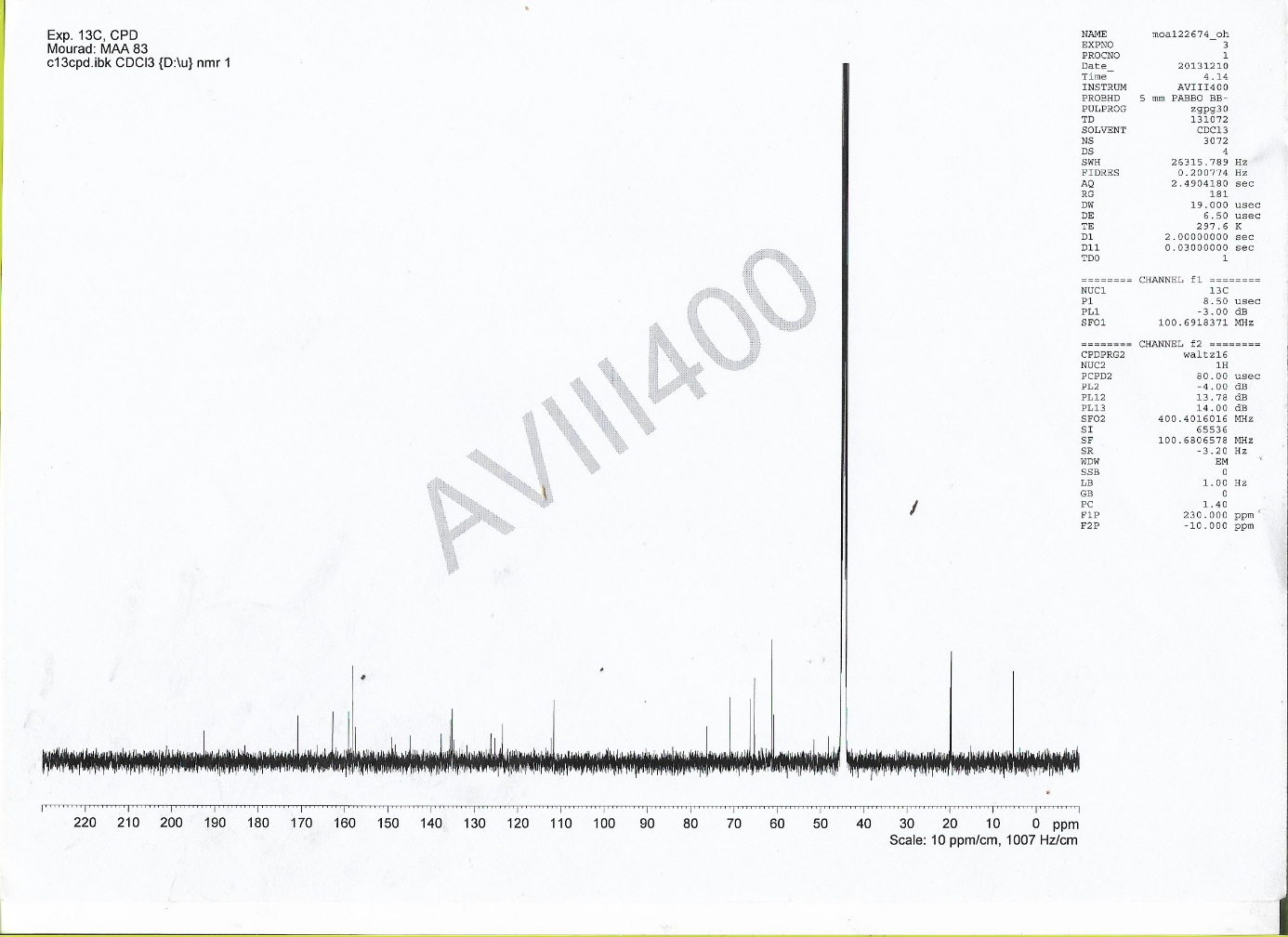


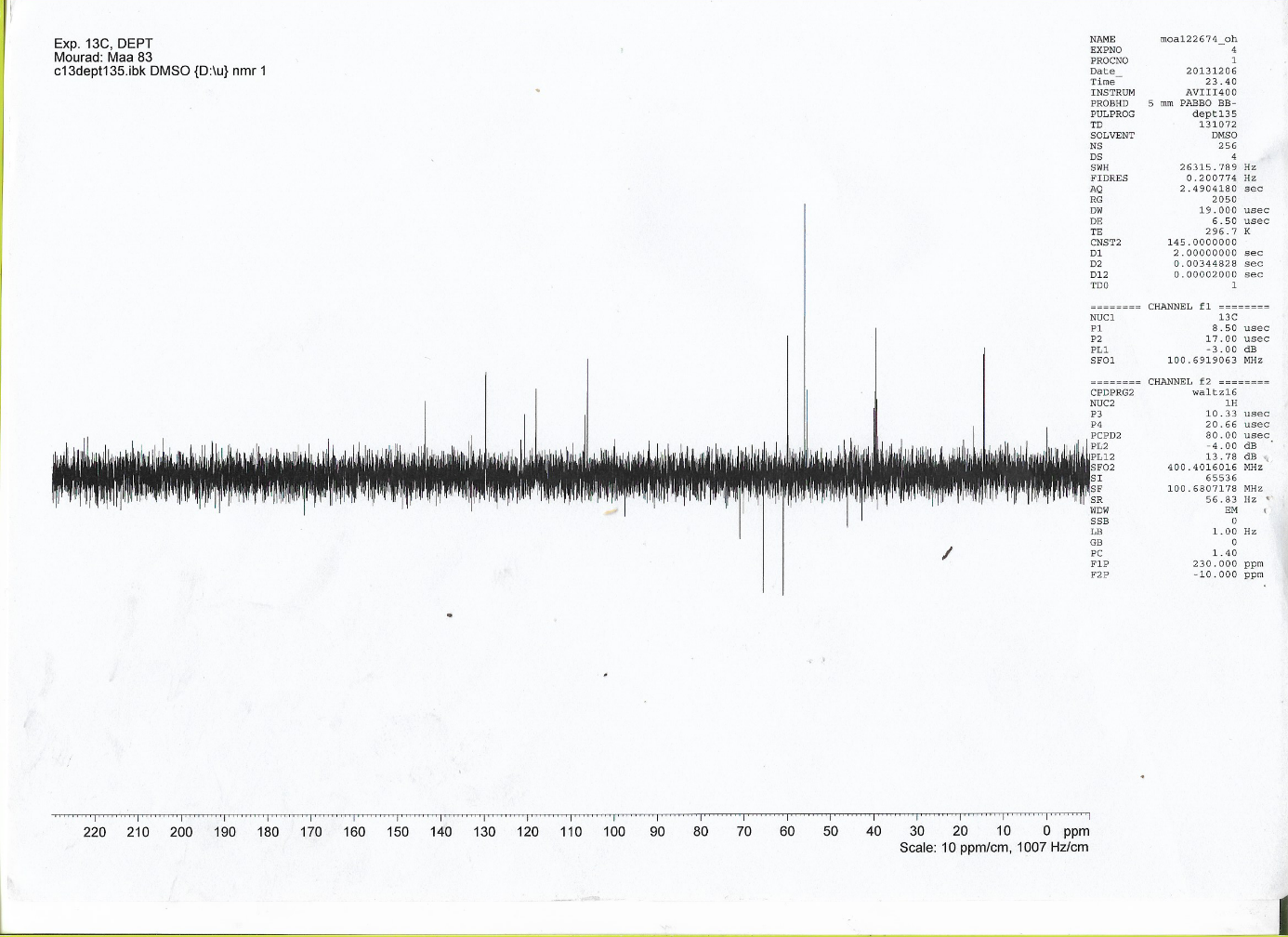

Supplement: Supplementary 3 — 1H NMR, 13C NMR, and DEPT. [file 4759821.f3.docx]
