# Supplementary material for: Synthesis, Anticancer Assessment, and Molecular Docking of Novel Chalcone-Thienopyrimidine Derivatives in HepG2 and MCF-7 Cell Lines
Source: Oxid Med Cell Longev. 2021 Dec 28;2021:4759821. doi: 10.1155/2021/4759821 (PMC8728392; doi:10.1155/2021/4759821)
Supplement: Supplementary 4 — ADME study and Tables S1–S18. [file 4759821.f4.docx]

**ADME study of compounds 3a-g, DRO, and venetoclax**

**Table S1: Physicochemical properties of 3a**


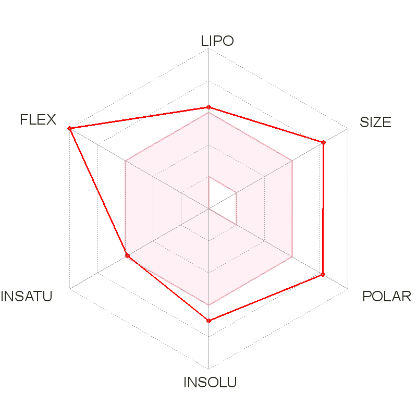


| SMILES | CCOC(=O)Cn1c(SCC(=O)Nc2ccc(cc2)C(=O)/C=C\c2ccccc2Cl)nc2c(c1=O)c1CCN(Cc1s2)C(=O)OCC |
| --- | --- |

| Physicochemical Properties | |
| --- | --- |
| Formula | C33H31ClN4O7S2 |
| Molecular weight | 695.20 g/mol |
| Num. heavy atoms | 47 |
| Num. arom. heavy atoms | 21 |
| Fraction Csp3 | 0.27 |
| Num. rotatable bonds | 15 |
| Num. H-bond acceptors | 8 |
| Num. H-bond donors | 1 |
| Molar Refractivity | 186.75 |
| TPSA [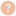](http://www.swissadme.ch/index.php) | 190.44 Å² |
| Lipophilicity | |
| Log *P*_o/w_ (iLOGP) [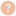](http://www.swissadme.ch/index.php) | 4.79 |
| Log *P*_o/w_ (XLOGP3) [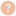](http://www.swissadme.ch/index.php) | 5.56 |
| Log *P*_o/w_ (WLOGP) [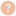](http://www.swissadme.ch/index.php) | 4.98 |
| Log *P*_o/w_ (MLOGP) [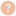](http://www.swissadme.ch/index.php) | 2.95 |
| Log *P*_o/w_ (SILICOS-IT) [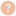](http://www.swissadme.ch/index.php) | 6.64 |
| Consensus Log *P*_o/w_ [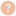](http://www.swissadme.ch/index.php) | 4.98 |
| Water Solubility | |
| Log *S* (ESOL) [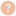](http://www.swissadme.ch/index.php) | -6.99 |
| Solubility | 7.05e-05 mg/ml ; 1.01e-07 mol/l |
| Class [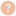](http://www.swissadme.ch/index.php) | Poorly soluble |
| Log *S* (Ali) [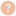](http://www.swissadme.ch/index.php) | -9.32 |
| Solubility | 3.33e-07 mg/ml ; 4.79e-10 mol/l |
| Class [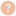](http://www.swissadme.ch/index.php) | Poorly soluble |
| Log *S* (SILICOS-IT) [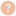](http://www.swissadme.ch/index.php) | -8.98 |
| Solubility | 7.26e-07 mg/ml ; 1.04e-09 mol/l |
| Class [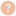](http://www.swissadme.ch/index.php) | Poorly soluble |
| Pharmacokinetics | |
| GI absorption [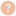](http://www.swissadme.ch/index.php) | Low |
| BBB permeant [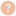](http://www.swissadme.ch/index.php) | No |
| P-gp substrate [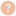](http://www.swissadme.ch/index.php) | No |
| CYP1A2 inhibitor [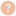](http://www.swissadme.ch/index.php) | No |
| CYP2C19 inhibitor [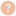](http://www.swissadme.ch/index.php) | No |
| CYP2C9 inhibitor [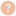](http://www.swissadme.ch/index.php) | Yes |
| CYP2D6 inhibitor [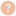](http://www.swissadme.ch/index.php) | Yes |
| CYP3A4 inhibitor [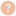](http://www.swissadme.ch/index.php) | Yes |
| Log *K*_p_ (skin permeation) [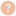](http://www.swissadme.ch/index.php) | -6.59 cm/s |
| Druglikeness | |
| Lipinski [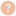](http://www.swissadme.ch/index.php) | No; 2 violations: MW>500, NorO>10 |
| Ghose [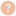](http://www.swissadme.ch/index.php) | No; 3 violations: MW>480, MR>130, #atoms>70 |
| Veber [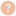](http://www.swissadme.ch/index.php) | No; 2 violations: Rotors>10, TPSA>140 |
| Egan [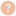](http://www.swissadme.ch/index.php) | No; 1 violation: TPSA>131.6 |
| Muegge [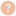](http://www.swissadme.ch/index.php) | No; 3 violations: MW>600, XLOGP3>5, TPSA>150 |
| Bioavailability Score [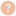](http://www.swissadme.ch/index.php) | 0.17 |
| Medicinal Chemistry | |
|  |  |
| PAINS [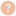](http://www.swissadme.ch/index.php) | 0 alert |
| Brenk [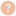](http://www.swissadme.ch/index.php) | 2 alerts: michael_acceptor_1, more_than_2_esters [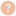](http://www.swissadme.ch/index.php) |
| Leadlikeness [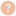](http://www.swissadme.ch/index.php) | No; 3 violations: MW>350, Rotors>7, XLOGP3>3.5 |
| Synthetic accessibility [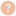](http://www.swissadme.ch/index.php) | 4.91 |

**Table S 2: Physicochemical properties of 3b**


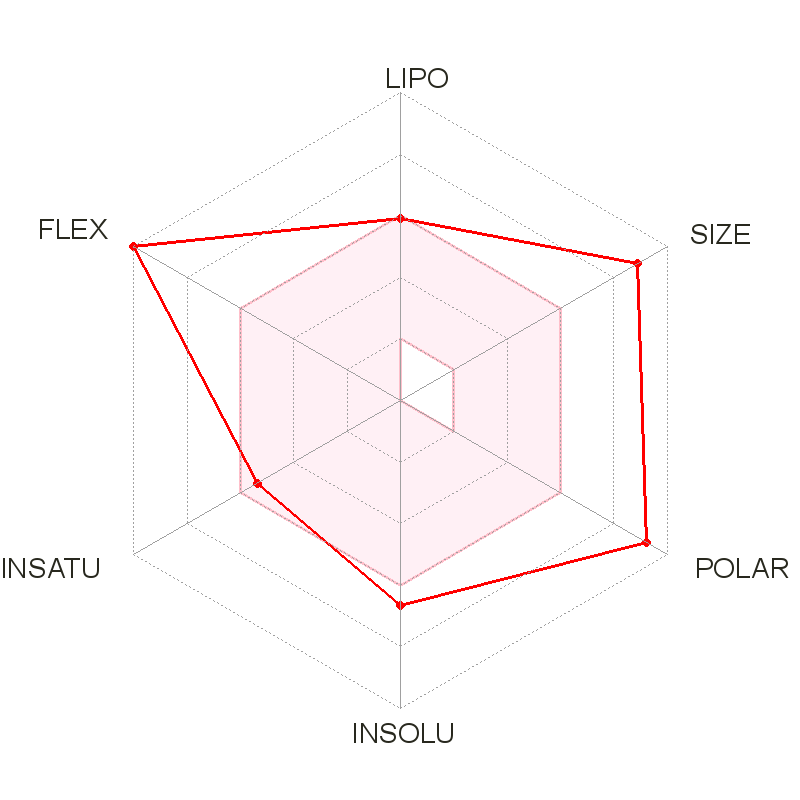


| SMILES | CCOC(=O)Cn1c(SCC(=O)Nc2ccc(cc2)C(=O)/C=C\c2cc(OC)c(c(c2)OC)OC)nc2c(c1=O)c1CCN(Cc1s2)C(=O)OCC |
| --- | --- |

| Physicochemical Properties | |
| --- | --- |
| Formula | C36H38N4O10S2 |
| Molecular weight | 750.84 g/mol |
| Num. heavy atoms | 52 |
| Num. arom. heavy atoms | 21 |
| Fraction Csp3 | 0.33 |
| Num. rotatable bonds | 18 |
| Num. H-bond acceptors | 11 |
| Num. H-bond donors | 1 |
| Molar Refractivity | 201.22 |
| TPSA [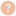](http://www.swissadme.ch/index.php) | 218.13 Å² |
| Lipophilicity | |
| Log *P*_o/w_ (iLOGP) [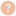](http://www.swissadme.ch/index.php) | 5.31 |
| Log *P*_o/w_ (XLOGP3) [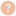](http://www.swissadme.ch/index.php) | 4.84 |
| Log *P*_o/w_ (WLOGP) [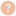](http://www.swissadme.ch/index.php) | 4.36 |
| Log *P*_o/w_ (MLOGP) [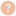](http://www.swissadme.ch/index.php) | 1.59 |
| Log *P*_o/w_ (SILICOS-IT) [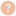](http://www.swissadme.ch/index.php) | 6.30 |
| Consensus Log *P*_o/w_ [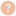](http://www.swissadme.ch/index.php) | 4.48 |
| Water Solubility | |
| Log *S* (ESOL) [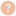](http://www.swissadme.ch/index.php) | -6.66 |
| Solubility | 1.66e-04 mg/ml ; 2.21e-07 mol/l |
| Class [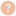](http://www.swissadme.ch/index.php) | Poorly soluble |
| Log *S* (Ali) [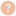](http://www.swissadme.ch/index.php) | -9.15 |
| Solubility | 5.26e-07 mg/ml ; 7.01e-10 mol/l |
| Class [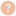](http://www.swissadme.ch/index.php) | Poorly soluble |
| Log *S* (SILICOS-IT) [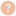](http://www.swissadme.ch/index.php) | -8.66 |
| Solubility | 1.63e-06 mg/ml ; 2.17e-09 mol/l |
| Class [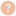](http://www.swissadme.ch/index.php) | Poorly soluble |
| Pharmacokinetics | |
| GI absorption [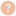](http://www.swissadme.ch/index.php) | Low |
| BBB permeant [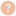](http://www.swissadme.ch/index.php) | No |
| P-gp substrate [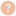](http://www.swissadme.ch/index.php) | No |
| CYP1A2 inhibitor [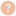](http://www.swissadme.ch/index.php) | No |
| CYP2C19 inhibitor [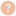](http://www.swissadme.ch/index.php) | No |
| CYP2C9 inhibitor [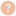](http://www.swissadme.ch/index.php) | Yes |
| CYP2D6 inhibitor [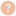](http://www.swissadme.ch/index.php) | Yes |
| CYP3A4 inhibitor [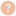](http://www.swissadme.ch/index.php) | Yes |
| Log *K*_p_ (skin permeation) [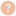](http://www.swissadme.ch/index.php) | -7.44 cm/s |
| Druglikeness | |
| Lipinski [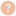](http://www.swissadme.ch/index.php) | No; 2 violations: MW>500, NorO>10 |
| Ghose [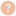](http://www.swissadme.ch/index.php) | No; 3 violations: MW>480, MR>130, #atoms>70 |
| Veber [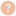](http://www.swissadme.ch/index.php) | No; 2 violations: Rotors>10, TPSA>140 |
| Egan [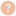](http://www.swissadme.ch/index.php) | No; 1 violation: TPSA>131.6 |
| Muegge [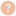](http://www.swissadme.ch/index.php) | No; 4 violations: MW>600, TPSA>150, Rotors>15, H-acc>10 |
| Bioavailability Score [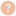](http://www.swissadme.ch/index.php) | 0.17 |
| Medicinal Chemistry | |
|  |  |
| PAINS [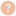](http://www.swissadme.ch/index.php) | 0 alert |
| Brenk [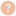](http://www.swissadme.ch/index.php) | 2 alerts: michael_acceptor_1, more_than_2_esters [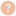](http://www.swissadme.ch/index.php) |
| Leadlikeness [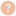](http://www.swissadme.ch/index.php) | No; 3 violations: MW>350, Rotors>7, XLOGP3>3.5 |
| Synthetic accessibility [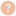](http://www.swissadme.ch/index.php) | 5.38 |

**Table S3: Physicochemical properties of 3c**


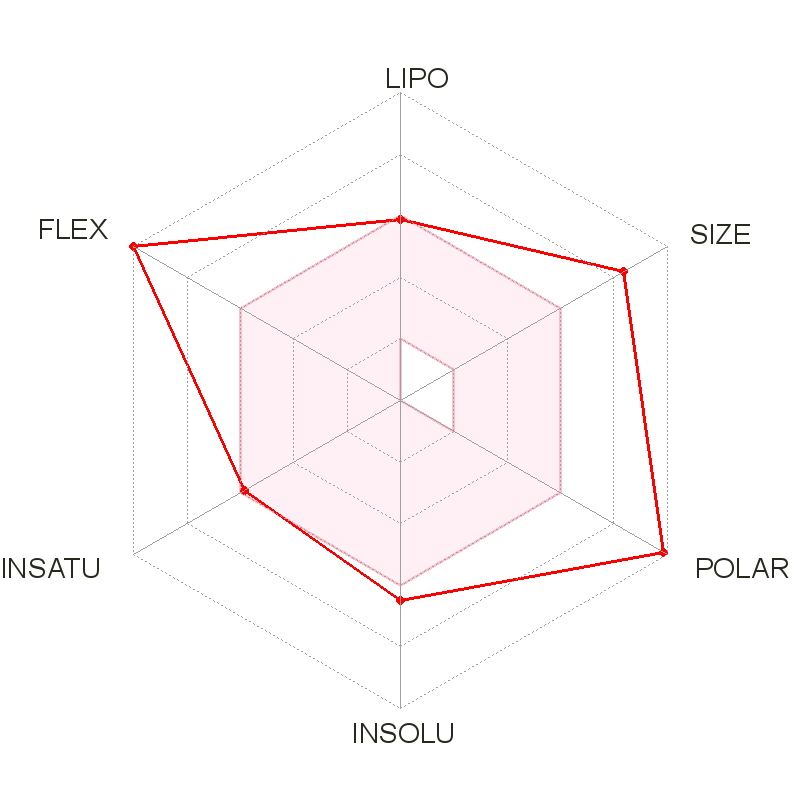


| SMILES | CCOC(=O)Cn1c(SCC(=O)Nc2ccc(cc2)C(=O)/C=C\c2cccc(c2)[N+](=O)[O-])nc2c(c1=O)c1CCN(Cc1s2)C(=O)OCC |
| --- | --- |

| Physicochemical Properties | |
| --- | --- |
| Formula | C33H31N5O9S2 |
| Molecular weight | 705.76 g/mol |
| Num. heavy atoms | 49 |
| Num. arom. heavy atoms | 21 |
| Fraction Csp3 | 0.27 |
| Num. rotatable bonds | 16 |
| Num. H-bond acceptors | 10 |
| Num. H-bond donors | 1 |
| Molar Refractivity | 190.56 |
| TPSA [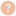](http://www.swissadme.ch/index.php) | 236.26 Å² |
| Lipophilicity | |
| Log *P*_o/w_ (iLOGP) [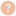](http://www.swissadme.ch/index.php) | 4.35 |
| Log *P*_o/w_ (XLOGP3) [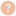](http://www.swissadme.ch/index.php) | 4.76 |
| Log *P*_o/w_ (WLOGP) [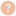](http://www.swissadme.ch/index.php) | 4.24 |
| Log *P*_o/w_ (MLOGP) [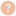](http://www.swissadme.ch/index.php) | 1.74 |
| Log *P*_o/w_ (SILICOS-IT) [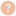](http://www.swissadme.ch/index.php) | 3.87 |
| Consensus Log *P*_o/w_ [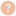](http://www.swissadme.ch/index.php) | 3.79 |
| Water Solubility | |
| Log *S* (ESOL) [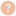](http://www.swissadme.ch/index.php) | -6.48 |
| Solubility | 2.36e-04 mg/ml ; 3.34e-07 mol/l |
| Class [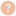](http://www.swissadme.ch/index.php) | Poorly soluble |
| Log *S* (Ali) [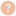](http://www.swissadme.ch/index.php) | -9.45 |
| Solubility | 2.49e-07 mg/ml ; 3.53e-10 mol/l |
| Class [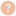](http://www.swissadme.ch/index.php) | Poorly soluble |
| Log *S* (SILICOS-IT) [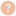](http://www.swissadme.ch/index.php) | -7.73 |
| Solubility | 1.32e-05 mg/ml ; 1.87e-08 mol/l |
| Class [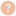](http://www.swissadme.ch/index.php) | Poorly soluble |
| Pharmacokinetics | |
| GI absorption [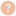](http://www.swissadme.ch/index.php) | Low |
| BBB permeant [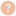](http://www.swissadme.ch/index.php) | No |
| P-gp substrate [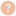](http://www.swissadme.ch/index.php) | No |
| CYP1A2 inhibitor [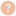](http://www.swissadme.ch/index.php) | No |
| CYP2C19 inhibitor [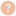](http://www.swissadme.ch/index.php) | No |
| CYP2C9 inhibitor [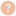](http://www.swissadme.ch/index.php) | Yes |
| CYP2D6 inhibitor [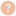](http://www.swissadme.ch/index.php) | No |
| CYP3A4 inhibitor [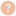](http://www.swissadme.ch/index.php) | Yes |
| Log *K*_p_ (skin permeation) [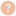](http://www.swissadme.ch/index.php) | -7.23 cm/s |
| Druglikeness | |
| Lipinski [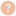](http://www.swissadme.ch/index.php) | No; 2 violations: MW>500, NorO>10 |
| Ghose [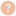](http://www.swissadme.ch/index.php) | No; 3 violations: MW>480, MR>130, #atoms>70 |
| Veber [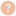](http://www.swissadme.ch/index.php) | No; 2 violations: Rotors>10, TPSA>140 |
| Egan [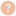](http://www.swissadme.ch/index.php) | No; 1 violation: TPSA>131.6 |
| Muegge [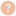](http://www.swissadme.ch/index.php) | No; 3 violations: MW>600, TPSA>150, Rotors>15 |
| Bioavailability Score [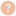](http://www.swissadme.ch/index.php) | 0.17 |
| Medicinal Chemistry | |
|  |  |
| PAINS [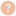](http://www.swissadme.ch/index.php) | 0 alert |
| Brenk [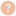](http://www.swissadme.ch/index.php) | 4 alerts: michael_acceptor_1, more_than_2_esters, nitro_group, oxygen-nitrogen_single_bond [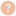](http://www.swissadme.ch/index.php) |
| Leadlikeness | No; 3 violations: MW>350, Rotors>7, XLOGP3>3.5 |
| Synthetic accessibility | 5.01 |

**Table S4: Physicochemical properties of 3d**

| SMILES | CCOCc1nc2sc3c(c2c(=O)n1CC(=O)Nc1ccc(cc1)C(=O)/C=C\c1ccc(c(c1OC)OC)OC)CCN(C3)C(=O)OCC |
| --- | --- |

| Physicochemical Properties | |
| --- | --- |
| Formula | C35H38N4O9S |
| Molecular weight | 690.76 g/mol |
| Num. heavy atoms | 49 |
| Num. arom. heavy atoms | 21 |
| Fraction Csp3 | 0.34 |
| Num. rotatable bonds | 16 |
| Num. H-bond acceptors | 10 |
| Num. H-bond donors | 1 |
| Molar Refractivity | 189.45 |
| TPSA | 175.76 Å² |
| Lipophilicity | |
| Log *P*_o/w_ (iLOGP) | 5.29 |
| Log *P*_o/w_ (XLOGP3) | 3.86 |
| Log *P*_o/w_ (WLOGP) | 4.09 |
| Log *P*_o/w_ (MLOGP) | 1.15 |
| Log *P*_o/w_ (SILICOS-IT) | 6.21 |
| Consensus Log *P*_o/w_ | 4.12 |
| Water Solubility | |
| Log *S* (ESOL) | -5.82 |
| Solubility | 1.06e-03 mg/ml ; 1.53e-06 mol/l |
| Class | Moderately soluble |
| Log *S* (Ali) | -7.25 |
| Solubility | 3.91e-05 mg/ml ; 5.65e-08 mol/l |
| Class | Poorly soluble |
| Log *S* (SILICOS-IT) | -8.70 |
| Solubility | 1.39e-06 mg/ml ; 2.01e-09 mol/l |
| Class | Poorly soluble |
| Pharmacokinetics | |
| GI absorption | Low |
| BBB permeant | No |
| P-gp substrate | No |
| CYP1A2 inhibitor | No |
| CYP2C19 inhibitor | No |
| CYP2C9 inhibitor | Yes |
| CYP2D6 inhibitor | Yes |
| CYP3A4 inhibitor | Yes |
| Log *K*_p_ (skin permeation) | -7.77 cm/s |
| Druglikeness | |
| Lipinski | No; 2 violations: MW>500, NorO>10 |
| Ghose | No; 3 violations: MW>480, MR>130, #atoms>70 |
| Veber | No; 2 violations: Rotors>10, TPSA>140 |
| Egan | No; 1 violation: TPSA>131.6 |
| Muegge | No; 3 violations: MW>600, TPSA>150, Rotors>15 |
| Bioavailability Score | 0.17 |
| Medicinal Chemistry | |
|  |  |
| PAINS | 0 alert |
| Brenk | 1 alert: michael_acceptor_1 |
| Leadlikeness | No; 3 violations: MW>350, Rotors>7, XLOGP3>3.5 |
| Synthetic accessibility | 5.26 |

**Table S5: Physicochemical properties of 3e**

| SMILES | CCOC(=O)N1CCc2c(C1)sc1c2c(=O)[nH]c(n1)SCC(=O)Nc1ccc(cc1)C(=O)/C=C\c1cc(OC)c(c(c1)OC)OC |
| --- | --- |

| Physicochemical Properties | |
| --- | --- |
| Formula | C32H32N4O8S2 |
| Molecular weight | 664.75 g/mol |
| Num. heavy atoms | 46 |
| Num. arom. heavy atoms | 21 |
| Fraction Csp3 | 0.28 |
| Num. rotatable bonds | 14 |
| Num. H-bond acceptors | 9 |
| Num. H-bond donors | 2 |
| Molar Refractivity | 180.61 |
| TPSA | 202.69 Å² |
| Lipophilicity | |
| Log *P*_o/w_ (iLOGP) | 4.31 |
| Log *P*_o/w_ (XLOGP3) | 4.37 |
| Log *P*_o/w_ (WLOGP) | 4.32 |
| Log *P*_o/w_ (MLOGP) | 1.61 |
| Log *P*_o/w_ (SILICOS-IT) | 6.43 |
| Consensus Log *P*_o/w_ | 4.21 |
| Water Solubility | |
| Log *S* (ESOL) | -6.13 |
| Solubility | 4.95e-04 mg/ml ; 7.44e-07 mol/l |
| Class | Poorly soluble |
| Log *S* (Ali) | -8.34 |
| Solubility | 3.02e-06 mg/ml ; 4.55e-09 mol/l |
| Class | Poorly soluble |
| Log *S* (SILICOS-IT) | -8.73 |
| Solubility | 1.23e-06 mg/ml ; 1.85e-09 mol/l |
| Class | Poorly soluble |
| Pharmacokinetics | |
| GI absorption | Low |
| BBB permeant | No |
| P-gp substrate | No |
| CYP1A2 inhibitor | No |
| CYP2C19 inhibitor | No |
| CYP2C9 inhibitor | Yes |
| CYP2D6 inhibitor | No |
| CYP3A4 inhibitor | Yes |
| Log *K*_p_ (skin permeation) | -7.25 cm/s |
| Druglikeness | |
| Lipinski | No; 2 violations: MW>500, NorO>10 |
| Ghose | No; 3 violations: MW>480, MR>130, #atoms>70 |
| Veber | No; 2 violations: Rotors>10, TPSA>140 |
| Egan | No; 1 violation: TPSA>131.6 |
| Muegge | No; 2 violations: MW>600, TPSA>150 |
| Bioavailability Score | 0.17 |
| Medicinal Chemistry | |
|  |  |
| PAINS | 0 alert |
| Brenk | 1 alert: michael_acceptor_1 |
| Leadlikeness | No; 3 violations: MW>350, Rotors>7, XLOGP3>3.5 |
| Synthetic accessibility | 4.78 |

**Table S 6: Physicochemical properties of 3f**

| SMILES | CCOCc1nc2sc3c(c2c(=O)n1CC(=O)Nc1ccc(cc1)C(=O)/C=C\c1cccc(c1)[N+](=O)[O-])CCN(C3)C(=O)OCC |
| --- | --- |

| Physicochemical Properties | |
| --- | --- |
| Formula | C32H31N5O8S |
| Molecular weight | 645.68 g/mol |
| Num. heavy atoms | 46 |
| Num. arom. heavy atoms | 21 |
| Fraction Csp3 | 0.28 |
| Num. rotatable bonds | 14 |
| Num. H-bond acceptors | 9 |
| Num. H-bond donors | 1 |
| Molar Refractivity | 178.80 |
| TPSA | 193.89 Å² |
| Lipophilicity | |
| Log *P*_o/w_ (iLOGP) | 3.21 |
| Log *P*_o/w_ (XLOGP3) | 3.78 |
| Log *P*_o/w_ (WLOGP) | 3.97 |
| Log *P*_o/w_ (MLOGP) | 1.55 |
| Log *P*_o/w_ (SILICOS-IT) | 3.78 |
| Consensus Log *P*_o/w_ | 3.26 |
| Water Solubility | |
| Log *S* (ESOL) | -5.64 |
| Solubility | 1.48e-03 mg/ml ; 2.30e-06 mol/l |
| Class | Moderately soluble |
| Log *S* (Ali) | -7.55 |
| Solubility | 1.84e-05 mg/ml ; 2.85e-08 mol/l |
| Class | Poorly soluble |
| Log *S* (SILICOS-IT) | -7.76 |
| Solubility | 1.13e-05 mg/ml ; 1.75e-08 mol/l |
| Class | Poorly soluble |
| Pharmacokinetics | |
| GI absorption | Low |
| BBB permeant | No |
| P-gp substrate | No |
| CYP1A2 inhibitor | No |
| CYP2C19 inhibitor | No |
| CYP2C9 inhibitor | Yes |
| CYP2D6 inhibitor | No |
| CYP3A4 inhibitor | Yes |
| Log *K*_p_ (skin permeation) | -7.55 cm/s |
| Druglikeness | |
| Lipinski | No; 2 violations: MW>500, NorO>10 |
| Ghose | No; 3 violations: MW>480, MR>130, #atoms>70 |
| Veber | No; 2 violations: Rotors>10, TPSA>140 |
| Egan | No; 1 violation: TPSA>131.6 |
| Muegge | No; 2 violations: MW>600, TPSA>150 |
| Bioavailability Score | 0.17 |
| Medicinal Chemistry | |
|  |  |
| PAINS | 0 alert |
| Brenk | 3 alerts: michael_acceptor_1, nitro_group, oxygen-nitrogen_single_bond |
| Leadlikeness | No; 3 violations: MW>350, Rotors>7, XLOGP3>3.5 |
| Synthetic accessibility | 4.86 |

**Table S7: Physicochemical properties of** **3g**

| SMILES | CCOC(=O)Cn1c(SCC(=O)Nc2ccc(cc2)C(=O)/C=C\c2cccc(c2)[N+](=O)[O-])nc2c(c1=O)c1CC(C)(C)NC(c1s2)(C)C |
| --- | --- |

| Physicochemical Properties | | |
| --- | --- | --- |
| Formula | C34H35N5O7S2 |  |
| Molecular weight | 689.80 g/mol |  |
| Num. heavy atoms | 48 |  |
| Num. arom. heavy atoms | 21 |  |
| Fraction Csp3 | 0.32 |  |
| Num. rotatable bonds | 13 |  |
| Num. H-bond acceptors | 9 |  |
| Num. H-bond donors | 2 |  |
| Molar Refractivity | 193.52 |  |
| TPSA | 218.75 Å² |  |
| Lipophilicity | | |
| Log *P*_o/w_ (iLOGP) | 4.62 |  |
| Log *P*_o/w_ (XLOGP3) | 5.27 |  |
| Log *P*_o/w_ (WLOGP) | 4.93 |  |
| Log *P*_o/w_ (MLOGP) | 1.85 |  |
| Log *P*_o/w_ (SILICOS-IT) | 5.27 |  |
| Consensus Log *P*_o/w_ | 4.39 |  |
| Water Solubility | | |
| Log *S* (ESOL) | -6.90 |  |
| Solubility | 8.63e-05 mg/ml ; 1.25e-07 mol/l |  |
| Class | Poorly soluble |  |
| Log *S* (Ali) | -9.61 |  |
| Solubility | 1.68e-07 mg/ml ; 2.43e-10 mol/l |  |
| Class | Poorly soluble |  |
| Log *S* (SILICOS-IT) | -9.51 |  |
| Solubility | 2.12e-07 mg/ml ; 3.07e-10 mol/l |  |
| Class | Poorly soluble |  |
| Pharmacokinetics | | |
| GI absorption | Low |  |
| BBB permeant | No |  |
| P-gp substrate | No |  |
| CYP1A2 inhibitor | No |  |
| CYP2C19 inhibitor | No |  |
| CYP2C9 inhibitor | No |  |
| CYP2D6 inhibitor | No |  |
| CYP3A4 inhibitor | Yes |  |
| Log *K*_p_ (skin permeation) | -6.77 cm/s |  |
| Drug-likeness | | |
| Lipinski | No; 2 violations: MW>500, NorO>10 |  |
| Ghose | No; 3 violations: MW>480, MR>130, #atoms>70 |  |
| Veber | No; 2 violations: Rotors>10, TPSA>140 |  |
| Egan | No; 1 violation: TPSA>131.6 |  |
| Muegge | No; 3 violations: MW>600, XLOGP3>5, TPSA>150 |  |
| Bioavailability Score | 0.17 |  |
| Medicinal Chemistry | | |
|  |  |  |
| PAINS | 0 alert |  |
| Brenk | 3 alerts: michael_acceptor_1, nitro_group, oxygen-nitrogen_single_bond |  |
| Leadlikeness | No; 3 violations: MW>350, Rotors>7, XLOGP3>3.5 |  |
| Synthetic accessibility | 5.09 |  |

Top of Form

Bottom of Form

Top of Form

Bottom of Form

Top of Form

Bottom of Form

Top of Form

Bottom of Form

**Table S8: Physicochemical properties of DRO**

| SMILES | NC[C@@H]1Cc2ccccc2CN1C(=O)c1ccccc1n1nc(c(c1C)Cl)C(=O)N(c1ccccc1)c1ccccc1 |
| --- | --- |

| Physicochemical Properties | |
| --- | --- |
| Formula | C34H30ClN5O2 |
| Molecular weight | 576.09 g/mol |
| Num. heavy atoms | 42 |
| Num. arom. heavy atoms | 29 |
| Fraction Csp3 | 0.15 |
| Num. rotatable bonds | 8 |
| Num. H-bond acceptors | 4 |
| Num. H-bond donors | 1 |
| Molar Refractivity | 169.56 |
| TPSA | 84.46 Å² |
| Lipophilicity | |
| Log *P*_o/w_ (iLOGP) | 3.71 |
| Log *P*_o/w_ (XLOGP3) | 5.94 |
| Log *P*_o/w_ (WLOGP) | 5.81 |
| Log *P*_o/w_ (MLOGP) | 4.76 |
| Log *P*_o/w_ (SILICOS-IT) | 4.81 |
| Consensus Log *P*_o/w_ | 5.01 |
| Water Solubility | |
| Log *S* (ESOL) | -7.14 |
| Solubility | 4.20e-05 mg/ml ; 7.30e-08 mol/l |
| Class | Poorly soluble |
| Log *S* (Ali) | -7.49 |
| Solubility | 1.87e-05 mg/ml ; 3.24e-08 mol/l |
| Class | Poorly soluble |
| Log *S* (SILICOS-IT) | -10.43 |
| Solubility | 2.16e-08 mg/ml ; 3.75e-11 mol/l |
| Class | Insoluble |
| Pharmacokinetics | |
| GI absorption | High |
| BBB permeant | No |
| P-gp substrate | No |
| CYP1A2 inhibitor | No |
| CYP2C19 inhibitor | Yes |
| CYP2C9 inhibitor | Yes |
| CYP2D6 inhibitor | No |
| CYP3A4 inhibitor | Yes |
| Log *K*_p_ (skin permeation) | -5.60 cm/s |
| Druglikeness | |
| Lipinski | No; 2 violations: MW>500, MLOGP>4.15 |
| Ghose | No; 4 violations: MW>480, WLOGP>5.6, MR>130, #atoms>70 |
| Veber | Yes |
| Egan | Yes |
| Muegge | No; 1 violation: XLOGP3>5 |
| Bioavailability Score | 0.17 |
| Medicinal Chemistry | |
|  |  |
| PAINS | 0 alert |
| Brenk | 0 alert |
| Leadlikeness | No; 3 violations: MW>350, Rotors>7, XLOGP3>3.5 |
| Synthetic accessibility | 4.51 |

**Table S9: Physicochemical properties of venetoclax (LBM)**

| SMILES | Clc1ccc(cc1)C1=C(CCC(C1)(C)C)CN1CCN(CC1)c1ccc(c(c1)Oc1cnc2c(c1)cc[nH]2)C(=O)NS(=O)(=O)c1ccc(c(c1)[N+](=O)[O-])NCC1CCOCC1 |
| --- | --- |

| Physicochemical Properties | |
| --- | --- |
| Formula | C45H50ClN7O7S |
| Molecular weight | 868.44 g/mol |
| Num. heavy atoms | 61 |
| Num. arom. heavy atoms | 27 |
| Fraction Csp3 | 0.38 |
| Num. rotatable bonds | 14 |
| Num. H-bond acceptors | 9 |
| Num. H-bond donors | 3 |
| Molar Refractivity | 246.70 |
| TPSA | 183.09 Å² |
| Lipophilicity | |
| Log *P*_o/w_ (iLOGP) | 5.57 |
| Log *P*_o/w_ (XLOGP3) | 8.18 |
| Log *P*_o/w_ (WLOGP) | 8.79 |
| Log *P*_o/w_ (MLOGP) | 3.22 |
| Log *P*_o/w_ (SILICOS-IT) | 4.83 |
| Consensus Log *P*_o/w_ | 6.12 |
| Water Solubility | |
| Log *S* (ESOL) | -9.78 |
| Solubility | 1.44e-07 mg/ml ; 1.65e-10 mol/l |
| Class | Poorly soluble |
| Log *S* (Ali) | -11.88 |
| Solubility | 1.13e-09 mg/ml ; 1.30e-12 mol/l |
| Class | Insoluble |
| Log *S* (SILICOS-IT) | -13.35 |
| Solubility | 3.86e-11 mg/ml ; 4.45e-14 mol/l |
| Class | Insoluble |
| Pharmacokinetics | |
| GI absorption | Low |
| BBB permeant | No |
| P-gp substrate | Yes |
| CYP1A2 inhibitor | No |
| CYP2C19 inhibitor | No |
| CYP2C9 inhibitor | No |
| CYP2D6 inhibitor | No |
| CYP3A4 inhibitor | No |
| Log *K*_p_ (skin permeation) | -5.79 cm/s |
| Druglikeness | |
| Lipinski | No; 2 violations: MW>500, NorO>10 |
| Ghose | No; 4 violations: MW>480, WLOGP>5.6, MR>130, #atoms>70 |
| Veber | No; 2 violations: Rotors>10, TPSA>140 |
| Egan | No; 2 violations: WLOGP>5.88, TPSA>131.6 |
| Muegge | No; 4 violations: MW>600, XLOGP3>5, TPSA>150, #rings>7 |
| Bioavailability Score | 0.17 |
| Medicinal Chemistry | |
|  |  |
| PAINS | 0 alert |
| Brenk | 2 alerts: nitro_group, oxygen-nitrogen_single_bond |
| Leadlikeness | No; 3 violations: MW>350, Rotors>7, XLOGP3>3.5 |
| Synthetic accessibility | 6.05 |

## **Table S 10: Molecular Properties and Drug-likeness of compound 3a**

|  | **Molecular formula:** C33 H31 Cl N4 O7 S2 **Molecular weight:** 694.13 (> 500) **Number of HBA:** 10 **Number of HBD:** 1 **MolLogP :** 5.33 (> 5) **MolLogS :** -5.68 (in Log(moles/L)) 1.45 (in mg/L) **MolPSA :** 104.86 A^2^ **MolVol :** 683.62 A^3^ **pKa of most Basic/Acidic group :** -2.45 / 11.81 **BBB Score :** 1.23 The Blood-Brain Barrier (BBB) Score: 6-High,0-Low *(DOI: 10.1021/acs.jmedchem.9b01220)* **Number of stereo centers:** 0 |
| --- | --- |

**Drug-likeness model score:** 1.01

## **Table S11: Molecular Properties and Drug-likeness of compound 3b**

| **Molecular formula:** C36 H38 N4 O10 S2 **Molecular weight:** 750.20 (> 500) **Number of HBA:** 13 (> 10) **Number of HBD:** 1 **MolLogP :** 4.57 **MolLogS :** -4.45 (in Log(moles/L)) 26.52 (in mg/L) **MolPSA :** 127.84 A^2^ **MolVol :** 764.06 A^3^ **pKa of most Basic/Acidic group :** -2.45 / 11.81 **BBB Score :** 0.69 The Blood-Brain Barrier (BBB) Score: 6-High,0-Low *(DOI: 10.1021/acs.jmedchem.9b01220)* **Number of stereo centers:** 0 |
| --- |

**Drug-likeness model score:** 1.35

## **Table S12: Molecular Properties and Drug-likeness of compound 3c**

| **Molecular formula:** C33 H31 N5 O9 S2 **Molecular weight:** 705.16 (> 500) **Number of HBA:** 12 (> 10) **Number of HBD:** 1 **MolLogP :** 4.79 **MolLogS :** -4.68 (in Log(moles/L)) 14.86 (in mg/L) **MolPSA :** 138.24 A^2^ **MolVol :** 693.94 A^3^ **pKa of most Basic/Acidic group :** -2.45 / 11.81 **BBB Score :** 0.69 The Blood-Brain Barrier (BBB) Score: 6-High,0-Low *(DOI: 10.1021/acs.jmedchem.9b01220)* **Number of stereo centers:** 0 |
| --- |

**Drug-likeness model score:** 0.72

## **Table S13: Molecular Properties and Drug-likeness of compound 3d**

| **Molecular formula:** C35 H38 N4 O9 S **Molecular weight:** 690.24 (> 500) **Number of HBA:** 11 (> 10) **Number of HBD:** 1 **MolLoSgP :** 3.77 **MolLogS :** -4.19 (in Log(moles/L)) 45.00 (in mg/L) **MolPSA :** 115.17 A^2^ **MolVol :** 720.46 A^3^ **pKa of most Basic/Acidic group :** -0.82 / 11.61 **BBB Score :** 1.08 The Blood-Brain Barrier (BBB) Score: 6-High,0-Low *(DOI: 10.1021/acs.jmedchem.9b01220)* **Number of stereo centers:** 0 |
| --- |

**Drug-likeness model score:** 1.42

##

## **Table S 14: Molecular Properties and Drug-likeness of compound 3e**

**Molecular formula:** C32 H32 N4 O8 S2
**Molecular weight:** 664.17 (> 500)
**Number of HBA:** 11 (> 10)
**Number of HBD:** 2
**MolLogP :** 4.23
**MolLogS :** -4.47 (in Log(moles/L)) 22.50 (in mg/L)
**MolPSA :** 116.14 A^2^
**MolVol :** 672.18 A^3^
**pKa of most Basic/Acidic group :** -2.15 / 10.94
**BBB Score :** 1.07 The Blood-Brain Barrier (BBB) Score: 6-High,0-Low *(DOI: 10.1021/acs.jmedchem.9b01220)*
**Number of stereo centers:** 0

**Drug-likeness model score:** 1.68

## **Table S15: Molecular Properties and Drug-likeness of compound 3f**

| **Molecular formula:** C32 H31 N5 O8 S **Molecular weight:** 645.19 (> 500) **Number of HBA:** 10 **Number of HBD:** 1 **MolLogP :** 3.98 **MolLogS :** -4.42 (in Log(moles/L)) 24.26 (in mg/L) **MolPSA :** 125.58 A^2^ **MolVol :** 650.41 A^3^ **pKa of most Basic/Acidic group :** -0.82 / 11.61 **BBB Score :** 0.69 The Blood-Brain Barrier (BBB) Score: 6-High,0-Low *(DOI: 10.1021/acs.jmedchem.9b01220)* **Number of stereo centers:** 0 |
| --- |

**Drug-likeness model score:** 0.82

## **Table S16: Molecular Properties and Drug-likeness of compound 3g**

| **Molecular formula:** C34 H35 N5 O7 S2 **Molecular weight:** 689.20 (> 500) **Number of HBA:** 11 (> 10) **Number of HBD:** 2 **MolLogP :** 5.33 (> 5) **MolLogS :** -5.26 (in Log(moles/L)) 3.81 (in mg/L) **MolPSA :** 126.81 A^2^ **MolVol :** 692.96 A^3^ **pKa of most Basic/Acidic group :** 6.69 / 11.81 **BBB Score :** 1.07 The Blood-Brain Barrier (BBB) Score: 6-High,0-Low *(DOI: 10.1021/acs.jmedchem.9b01220)* **Number of stereo centers:** 0 |
| --- |

**Drug-likeness model score:** 0.17

## **Table S17: Molecular Properties and Drug-likeness of DRO**

| **Molecular formula:** C34 H30 Cl N5 O2 **Molecular weight:** 575.21 (> 500) **Number of HBA:** 4 **Number of HBD:** 2 **MolLogP :** 4.52 **MolLogS :** -4.73 (in Log(moles/L)) 10.59 (in mg/L) **MolPSA :** 67.04 A^2^ **MolVol :** 559.71 A^3^ **pKa of most Basic/Acidic group :** 8.77 / 17.79 **BBB Score :** 2.90 The Blood-Brain Barrier (BBB) Score: 6-High,0-Low *(DOI: 10.1021/acs.jmedchem.9b01220)* **Number of stereo centers:** 1 |
| --- |

**Drug-likeness model score:** 0.38

## **Table S18: Molecular Properties and Drug-likeness of Venetoclax**

| **Molecular formula:** C45 H50 Cl N7 O7 S **Molecular weight:** 867.32 (> 500) **Number of HBA:** 9 **Number of HBD:** 3 **MolLogP :** 8.27 (> 5) **MolLogS :** -6.53 (in Log(moles/L)) 0.25 (in mg/L) **MolPSA :** 140.98 A^2^ **MolVol :** 843.71 A^3^ **pKa of most Basic/Acidic group :** 5.75 / 3.83 **BBB Score :** 0.26 The Blood-Brain Barrier (BBB) Score: 6-High,0-Low *(DOI: 10.1021/acs.jmedchem.9b01220)* **Number of stereo centers:** 1 |
| --- |

**Drug-likeness model score:** 0.50
